# CCL3 is produced by aged neutrophils across cancers and promotes tumor growth

**DOI:** 10.1016/j.ccell.2026.01.006

**Published:** 2026-02-05

**Authors:** Evangelia Bolli, Pratyaksha Wirapati, Mehdi Hicham, Yuxuan Xie, Marie Siwicki, Florent Duval, Anne-Gaëlle Goubet, Máté Kiss, Béatrice Zitti, Thomas Zwahlen, Sheri Mcdowell, Ruben Bill, Simona Angerani, Camilla Engblom, Seth Anderson, Aiping Jiang, Oliver Hartley, David B. Sykes, Maja Jankovic, Nadine Fournier, Matthias Gunzer, David Tarussio, Stéphanie Tissot, Peter M. Sadow, William C. Faquin, Moshe Sade-Feldman, Ralph Weissleder, Sara Pai, François Mercier, Robert Manguso, Mikaël J. Pittet

**Affiliations:** 1 Department of Pathology and Immunology, Faculty of Medicine, University of Geneva, Geneva, Switzerland; 2 AGORA Cancer Research Center, and Swiss Cancer Center Leman, Lausanne, Switzerland; 3 Translational Research Center in Oncohaematology, University of Geneva, Geneva, Switzerland; 4 Snyder Institute for Chronic Diseases, University of Calgary, Calgary, Alberta, Canada; 5 Translational Data Science Group, Swiss Institute of Bioinformatics, Lausanne, Switzerland; 6 Bioinformatics Core Facility, Swiss Institute of Bioinformatics, Lausanne, Switzerland; 7 Department of Medical Oncology, Inselspital, Bern University Hospital, University of Bern, Bern, Switzerland; 8 SciLifeLab, Department of Medicine, Solna, Karolinska Institutet and Karolinska University Hospital, Stockholm, Sweden; 9 Broad Institute of Massachusetts Institute of Technology and Harvard, Cambridge, MA, USA; 10 Center for Cancer Research, Massachusetts General Hospital, Boston, MA, USA; 11 Mass General Brigham Cancer Center, Boston, MA, USA; 12 Lady Davis Institute for Medical Research, McGill University, Montréal, Quebec, Canada; 13 Institute for Experimental Immunology and Imaging, University Hospital Essen, University of Duisburg-Essen, Essen, Germany; 14 Leibniz-Institut für Analytische Wissenschaften ISAS -e.V., Dortmund, Germany; 15 Department of Oncology, Lausanne University Hospital (CHUV), Lausanne, Switzerland; 16 Department of Surgery, Mass General Brigham, Harvard Medical School, Boston, Massachusetts, USA; 17 Department of Medicine, Massachusetts General Hospital, Boston, MA, USA; 18 Center for Systems Biology, Massachusetts General Hospital, Cambridge St, Boston, MA, USA; 19 Department of Radiology, Massachusetts General Hospital, Fruit St, Boston, MA, USA; 20 Department of Systems Biology, Harvard Medical School, Longwood Ave, Boston, MA, USA; 21 Department of Surgery, Yale University School of Medicine, New Haven, Connecticut, USA; 22 Ludwig Institute for Cancer Research, Lausanne Branch, Lausanne, Switzerland; 23 Department of Oncology, Geneva University Hospitals (HUG), Geneva, Switzerland; 24 These authors contributed equally; 25 Senior author; 26 Lead contact

## Abstract

Tumor-associated neutrophils (TANs) are abundant across cancers, yet their phenotypic diversity and functional states remain poorly defined. Here, we introduce a cell-type probability classifier that recovers low-transcript neutrophils from scRNAseq datasets, enabling pan-cancer analyses of TAN heterogeneity. Across >190 human and murine tumors, we identify a conserved differentiation trajectory that culminates in a terminal CCL3^hi^ state. This state exhibits pro-tumor transcriptional programs, including those involved in hypoxic adaptation and senescence. Consistently, CCL3^hi^ TANs are enriched in hypoxic tumor niches in both humans and mice. Through mechanistic perturbations of neutrophil-derived CCL3 in mice, we show that it sustains TAN survival in hypoxic tumor regions via CCR1-dependent signaling. These findings establish CCL3 as a conserved marker and functional driver of pro-tumor neutrophils in growing tumors, and provide a scalable framework for dissecting neutrophil biology across cancer types.

## In brief

Bolli et al. develop a classifier that rescues neutrophils typically lost in scRNA-seq data, enabling unified pancancer analyses. This approach identifies CCL3^hi^ aged tumor-associated neutrophils conserved across human and mouse cancers. Mechanistic perturbations show that neutrophil-derived CCL3 drives their CCR1-dependent survival in hypoxic niches, thereby promoting tumor progression.

## Graphical abstract

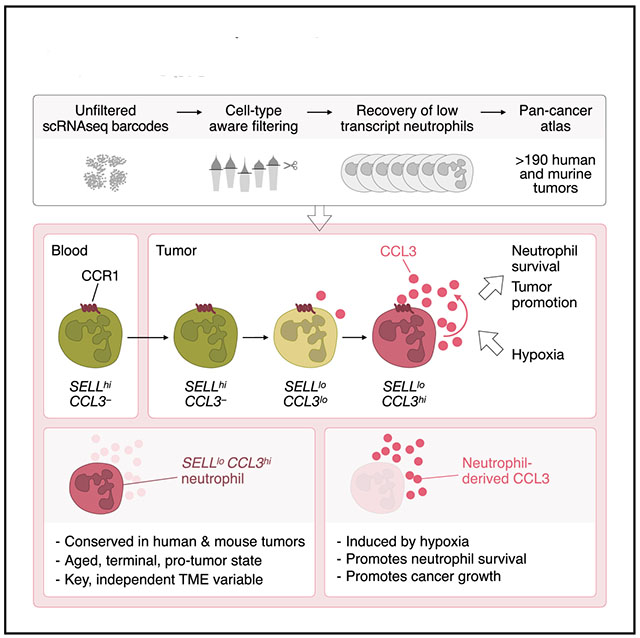

## INTRODUCTION

Neutrophils are the body’s first line of defense against injury and infection, yet they also play fundamental roles in cancer. High neutrophil abundance is reported across many human cancers and often correlates with poor outcomes.^1–11^ In mouse models, tumor-associated neutrophils (TANs) can promote tumor growth and modulate therapy responses.^2,4,12–24^ However, neutrophils are functionally heterogeneous,^17,25–37^ and only some appear to actively promote cancer.

Various markers of tumor-promoting neutrophils have been described, typically in specific cancer contexts. In mice, reported TAN subsets include SiglecF^hi^ cells in *Kras^G12D^;Tp53^null^* (KP)-driven lung tumors,^12^ dcTRAIL-R1^hi^ cells in *Pdx1^Cre^; Kras^G12D^;Trp53^R172H/+^* (KPC)-driven pancreatic ductal adenocarcinoma,^17^ CD71^+^ cells in GL261 and SB28 brain tumors,^37^ CD300LD^+^ cells in B16-F10 melanoma,^14^ and FATP2^+^ cells in several tumor models.^20^ In humans, LOX-1^+^ TANs have been identified in lung and head and neck cancers,^7^ CD71^+^ TANs in brain cancers,^37^ and CD300LD^+^ TANs^14^ and FATP2^+^ TANs^20^ in different cancer types. These observations suggest that tumor-promoting neutrophils are often defined by context-dependent markers, leaving unclear whether conserved neutrophil states and universal markers exist across tumors and species.

Large scRNAseq datasets could, in principle, resolve neutrophil heterogeneity, but neutrophils’ low transcriptional activity leads to their underrepresentation unless enrichment^31,35^ or platform optimization^32^ is used. This contrasts with histological and flow cytometry data, which often reveal substantial TAN infiltration.^32,38–40^ To address this matter, we developed a classifier capable of rescuing these cells from published datasets, enabling large-scale analyses of neutrophil states across human and murine cancers.

Another challenge in studying neutrophils is their resistance to genetic manipulation. Consequently, most mechanistic insights derive from broad depletion approaches,^2,34,41,42^ which preclude investigations of gene-specific contributions to neutrophil function. To overcome this limitation, we used two complementary strategies that preserve neutrophils while enabling their genetic manipulation: (1) a CRISPR/Cas9-based approach for neutrophil-specific gene knockout and (2) an ER-Hoxb8-based approach^43,44^ for gain- and loss-of-function studies.

Using this framework, we identify CCL3 as a conserved marker of terminally differentiated, pro-tumor neutrophils in growing human and mouse tumors. Targeted perturbation studies in mice show that neutrophil-derived CCL3 supports TAN survival in hypoxic tumor niches, thereby promoting tumor growth.

## RESULTS

### Cell type probability filtering identifies neutrophils in scRNAseq datasets

**Figure 1.**
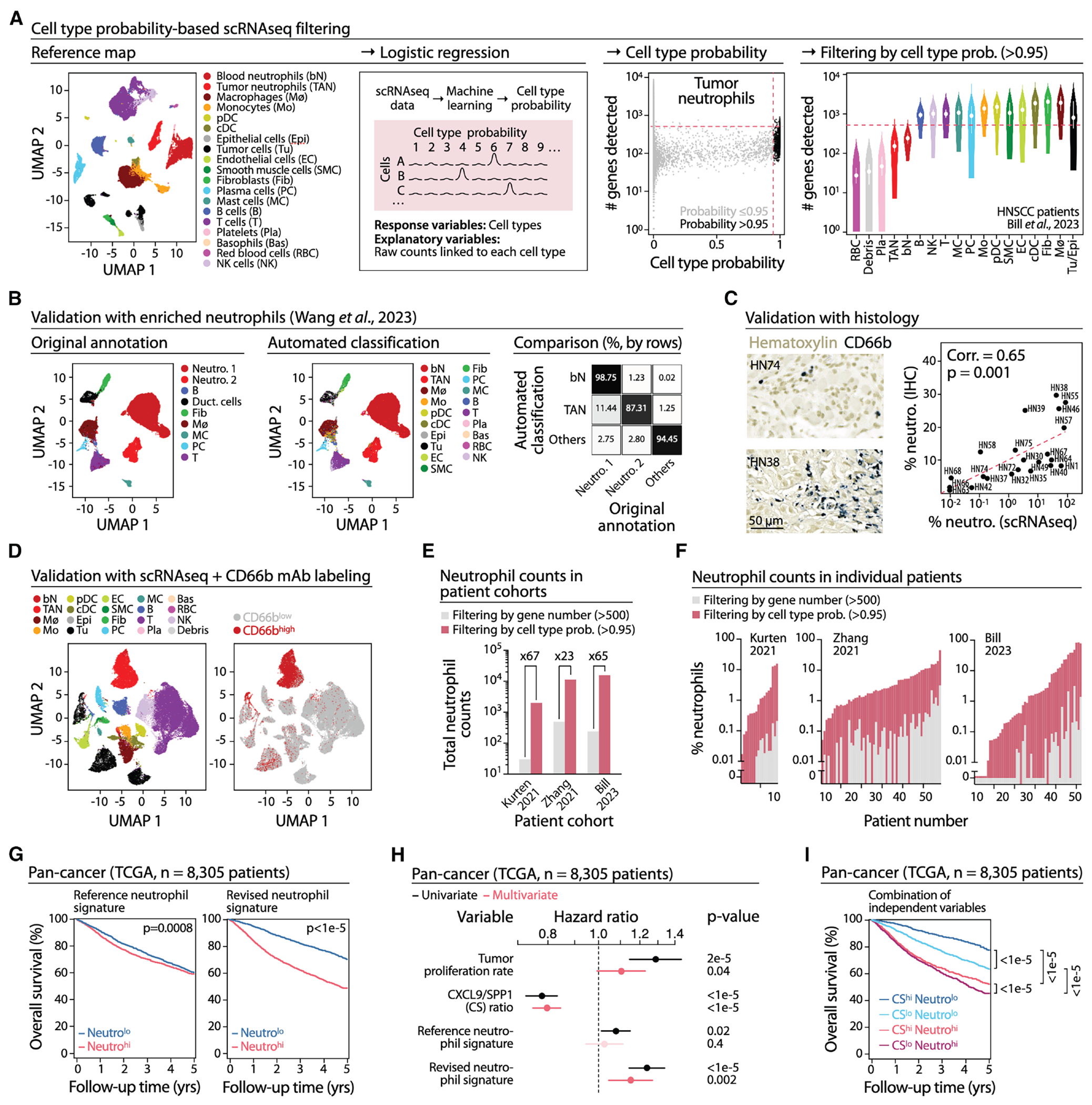
Cell type probability-based scRNAseq filtering identifies neutrophils as independent predictors of cancer outcome (A) Cell type probability-based scRNAseq classifier. From left to right: classifier trained on human blood and tumor scRNAseq data from Zilionis et al.^30^ (reference map shows uniform manifold approximation and projection (UMAP)); logistic regression used to assign each event a probability of belonging to a given cell type; example showing events classified as neutrophils (black dots) with their gene counts (500 gene cutoff in red); classifier-based identification of multiple cell types in tumor data from Bill et al.^45^ with corresponding gene counts (500 gene cutoff in red). (B) Original (left) and automated classifier (middle) annotations of neutrophil-enriched data from Wang et al.,^35^ with a direct comparison of the two approaches (right). (C) CD66b immunohistochemistry for neutrophil detection in HNSCC (left: two examples shown) and correlation between neutrophil content measured by immunohistochemistry and by the classifier on matched scRNAseq data (right, *n* = 22). Pearson’s correlation is shown. (D) Classifier-based detection of neutrophils and other cells in 11 HNSCC samples (left) and identification of CD66b mAb signal on the same cells (right). (E) Total neutrophil counts detected by the classifier (pink) versus standard gene-count filtering (gray) in three public datasets.^45–47^ (F) Same analysis as in (e), shown for individual tumors. (G) Kaplan-Meier plots of Neutro^hi^ vs. Neutro^lo^ tumors, defined using either a reference neutrophil signature (left) or a revised neutrophil signature based on our neutrophil abundance predictor (right), and applied to bulk RNAseq TCGA data (*n* = 8,305) from multiple cancer types. (H) Univariate (black) and multivariate (red) Cox regression analyses of four signatures: tumor proliferation rate,^48^ CS ratio,^45^ reference neutrophil signature,^48^ and our revised neutrophil signature. (I) Kaplan-Meier plot of TCGA data (*n* = 8,305) showing four patient groups defined by CS status (hi/lo) and neutrophil abundance (hi/lo).

We developed a cell classifier that infers cell identity directly from raw count matrices before any filtering is applied. Unlike fixed UMI or gene-count thresholds, the classifier models cell type-specific transcriptional properties using logistic regression with an added “debris” class generated by downsampling, enabling discrimination between true cells and low-quality profiles. Trained on an inDrop dataset^30^ that effectively captures neutrophils (Figure S1A), and applied to scRNAseq data from 52 head and neck squamous cell carcinoma (HNSCC) samples,^45^ the classifier identified neutrophils with <500 detected genes (i.e., cells excluded by common thresholds; Figure 1A), while preserving performance on high-transcript populations (Figure S1B). Whereas the original study recovered some neutrophils only through relaxed filtering and manual annotation, our approach captured them automatically.

We evaluated the classifier’s accuracy using complementary strategies. First, in neutrophil-optimized scRNAseq datasets (enriched PDAC neutrophils^35^ and enriched neutrophils from multiple cancers^25^), the classifier closely matched original annotations (Figures 1B, S1C, and S1D). Second, matched scRNA-seq and histological analyses from HNSCC samples showed strong concordance between classifier-based neutrophil abundance and CD66b staining (Figure 1C). Third, scRNAseq combined with CD66b mAb staining in HNSCC showed that the classifier-defined TAN cluster overlapped extensively with CD66b^+^ regions (Figure 1D).

We next compared the capacity of the classifier and conventional gene-count filters to recover neutrophils across multiple public datasets. In two HNSCC and one ESCC cohort,^45–47^ the classifier identified on average 65-, 67-, and 23-fold more neutrophils, respectively (Figures 1E, S1E, and S1F), and revealed substantial inter-patient variability (Figure 1F). It also recovered neutrophils from NSCLC^49^ samples (Figure S1G) in which neutrophils had not been reported, and it showed high concordance with annotations in a filtered HNSCC dataset^50^ (Figure S1H).

These results indicate that the classifier robustly detects neutrophils across cancer types and scRNAseq platforms, overcoming key limitations of conventional filters. It also enables persample processing of large, unfiltered matrices and provides cell-type-specific thresholding within a single regression model, removing the need for manual quality-control tuning.

### Neutrophils are independent predictors of cancer outcome

The classifier’s ability to recover previously overlooked TANs enabled us to reassess the prognostic relevance of TAN abundance across cancer types. We first generated a neutrophil abundance predictor based on the TAN pseudo-bulk transcriptional profile. Applied to bulk RNAseq data from TCGA (*n* = 8,305 patients), the predictor showed stronger prognostic performance than a widely used reference neutrophil signature^48^ (Figure 1G).

To examine robustness and independence of this prognostic signal, we performed Cox regression analyses to compare the revised and reference neutrophil signatures alongside two established prognostic variables: (i) CXCL9/SPP1 (CS) macrophage polarity,^45^ and (ii) tumor proliferation rate.^48^ All four variables were significant in univariate models, but in multivariate models only the revised neutrophil signature, CS macrophage polarity, and tumor proliferation rate remained significant, whereas the reference neutrophil signature did not (Figure 1H). Thus, neutrophil abundance captured by our predictor is an independent, pan-cancer prognostic TME variable, comparable to CS macrophage polarity.

We illustrate these findings using Kaplan-Meier analyses on the same TCGA cohort (Figure 1I). Patients were stratified into four groups based on CS ratio and predicted neutrophil abundance. Survival outcomes differed markedly across groups. Among patients with CS^hi^, high neutrophil abundance was associated with shorter survival compared to low neutrophil abundance (CS^hi^ Neutro^hi^ vs. CS^hi^ Neutro^lo^). Similarly, among patients with CS^lo^, high neutrophil abundance was again associated with worse outcomes (CS^lo^ Neutro^hi^ vs. CS^lo^ Neutro^lo^). These patterns underscore the independent prognostic contribution of TAN abundance.

These analyses show that the classifier reliably recovers TANs, even in datasets where they were previously reported as absent. By detecting neutrophils in larger numbers and across more patients, the classifier enables the systematic investigation of TAN heterogeneity using the extensive collection of existing scRNA-seq datasets, as detailed later in discussion.

Of note, in two immunotherapy trial datasets,^51^ the neutrophil abundance predictor was not linked to overall or progression-free survival (*p* = 0.48) and *p* = 0.99, respectively), whereas CS macrophage polarity was significantly associated with both endpoints (*p* = 0.013 and *p* = 0.00001). While additional analyses across larger cohorts are needed, these data suggest that, unlike CS macrophage polarity, TAN abundance alone may not be a strong determinant of immunotherapy outcome, while remaining a strong, independent predictor of pan-cancer prognosis.

### *CCL3^hi^* neutrophils mark an aged, pro-tumor state across human tumors

**Figure 2.**
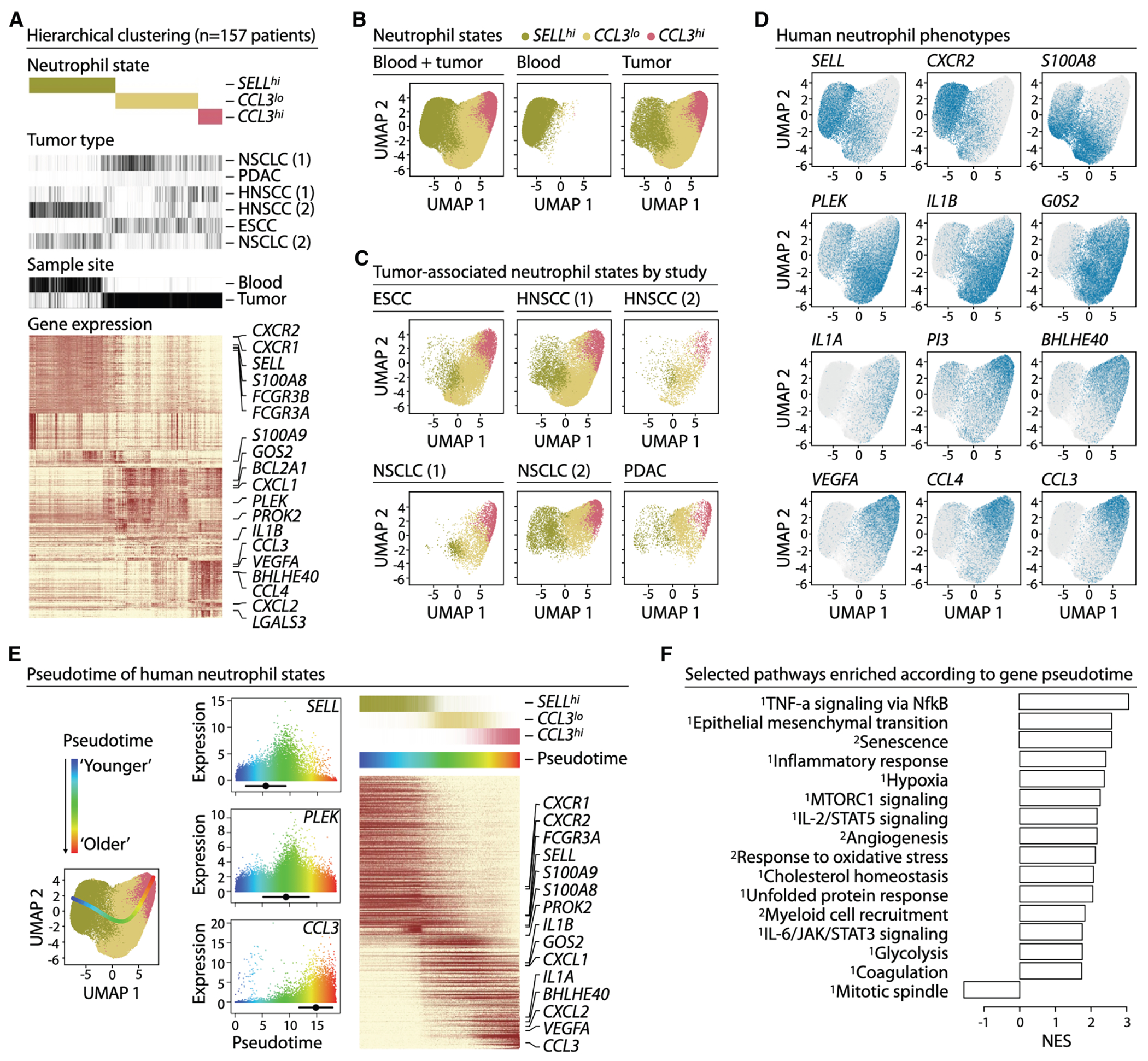
*CCL3*^*hi*^ neutrophils mark an aged, pro-tumor state across human tumors (A) Hierarchical clustering of 6 human cancer scRNAseq datasets^30,32,35,45–47^ (*n* = 157 patients, Table S1) filtered by cell type probability, including blood and tumor samples. Three primary neutrophil states were identified: a blood-like *SELL*^*hi*^, *CCL3*^*lo*^, and *CCL3*^*hi*^ TAN. (B and C) UMAPs of combined (left), blood-only (middle), tumor-only (right) (b), and per study (c) neutrophil heterogeneity. (D) UMAP expression pattern of some example genes. (E) Pseudotime computed using a principal curve fit to data from panel b, starting from “young” (*SELL*^*hi*^) to the most terminal “old” state (*CCL3*^*hi*^) (left). The expression-weighted mean pseudotime and its standard deviation are summarized for each gene. A representative gene (*SELL*, *PLEK*, *CCL3*) is shown for each neutrophil state (middle). Heatmap displaying pseudotime-ordered cells and genes (right). (F) Genes ordered by pseudotime were used in gene set enrichment analysis (GSEA) against MSigDB hallmark gene sets^52^ (identified with the sign “^1^”) and curated tumor neutrophil gene sets^12,26,53^ (identified with the sign “^2^”). NES; Normalized Enrichment Score.

To examine neutrophil heterogeneity across human tumors, we analyzed scRNAseq datasets from ESCC, HNSCC, NSCLC, and PDAC^30,32,35,45–47^ (Table S1), including some with matched blood samples. Using the classifier, we identified neutrophils in all datasets. Hierarchical clustering revealed mixed clusters containing neutrophils from every cohort, indicating robust integration and shared neutrophil states across cancers (Figure 2A). Tumor-associated neutrophils were transcriptionally distinct from blood neutrophils, reflecting tumor-driven adaptation.

Neutrophils formed a continuous spectrum of states, consistent with previous observations,^30^ which resolved into three primary subsets: *SELL^hi^*, *CCL3^lo^*, and *CCL3^hi^* (Figures 2A–2C). *SELL^hi^* neutrophils expressed naive/circulating phenotypes such as *SELL*, *CXCR2*, *CXCR1*, and S100A8 (Figures 2A, 2D, and S2A) and were enriched in blood (Figure 2B). *CCL3^lo^* and *CCL3^hi^* neutrophils were tumor-enriched and expressed *PLEK, IL1B and G0S2* (Figures 2A, 2D, and S2A). *CCL3^hi^* neutrophils showed further upregulation of *CCL4, VEGFA*, *BHLHE40* and *PI3* (Figures 2A, 2D, and S2A). Some of these genes have been associated with pro-tumor programs.^17,30,35,36^ Other reported pro-tumor neutrophil markers,^7,14,20,37^ including *OLR1* (encoding LOX-1), *TFRC* (encoding CD71), *SLC27A2* (encoding FATP2), and *CD300LD*, were expressed inconsistently across cancers and at lower levels than *CCL3* (Figure S2B), highlighting *CCL3* as a more robust and conserved marker of human TANs.

Gene expression changes across states were gradual (Figures 2A, 2D, and S2A), suggesting a continuous trajectory of differentiation. We modeled this progression using a principal curve to estimate pseudotime, revealing a trajectory from “young” *SELL^hi^* neutrophils to “old” *CCL3^hi^* neutrophils (Figure 2E). This directionality was supported by declining expression of cell cycle genes (mitotic spindle) and increasing expression of senescence-associated genes^53^ along pseudotime (Figure 2F). CytoTRACE2^54^ independently confirmed this ordering: all neutrophils fell within the differentiated end of the potency spectrum, with *SELL^hi^* cells consistently scoring higher than *CCL3^hi^* cells across datasets (Figure S2C).

We next summarized dynamic gene expression changes across pseudotime (Figures 2E and S2D). Gene set enrichment^12,26,52,53^ revealed that pathways increasing along pseudotime, i.e., enriched in *CCL3^hi^* neutrophils, were predominantly associated with pro-tumor programs, such as senescence, hypoxia, and response to oxidative stress (Figure 2F). These features were reproducible across cohorts, indicating shared biology rather than dataset-specific effects.

This three-state continuum aligned well with previously reported TAN subtypes^25,30,35^ (Figure S3A). For example, NEUTRO1 and NEUTRO2 from Wang et al.^35^ corresponded to *SELL^hi^* and *CCL3^hi^* states, respectively, and the ten subsets from Wu et al.^25^ mapped along the same trajectory. Context-dependent programs described in prior studies also fit within this framework. For example, *CXCR2*^+^ blood-associated neutrophils^55^ localized to the *SELL^hi^* region and distinguished circulating from tumor neutrophils (Figure 2D), while ISG^+^ neutrophils^26^ positioned along the *SELL^hi^*→*CCL3^hi^* continuum rather than forming an independent branch (Figure S3B).

In summary, our analysis of neutrophils across multiple human cancer types indicates a conserved continuum of TAN states shared across patients and tumor types, with additional context-dependent programs that can exist on top of these shared states. Within this trajectory, *CCL3^hi^* TANs represent a terminal, aged state enriched for pro-tumor transcriptional programs, and CCL3 emerges as a robust and conserved marker of these cells.

### *Ccl3^hi^* neutrophils mark an aged, pro-tumor state across mouse tumors

Mice are a primary model for mechanistic studies, making it important to evaluate how well murine neutrophil states mirror those in humans. Using the same classifier capable of detecting low-transcript cells, we analyzed scRNAseq data from 39 mice across 9 orthotopic tumor models^30,56–62^ (Table S2).

**Figure 3.**
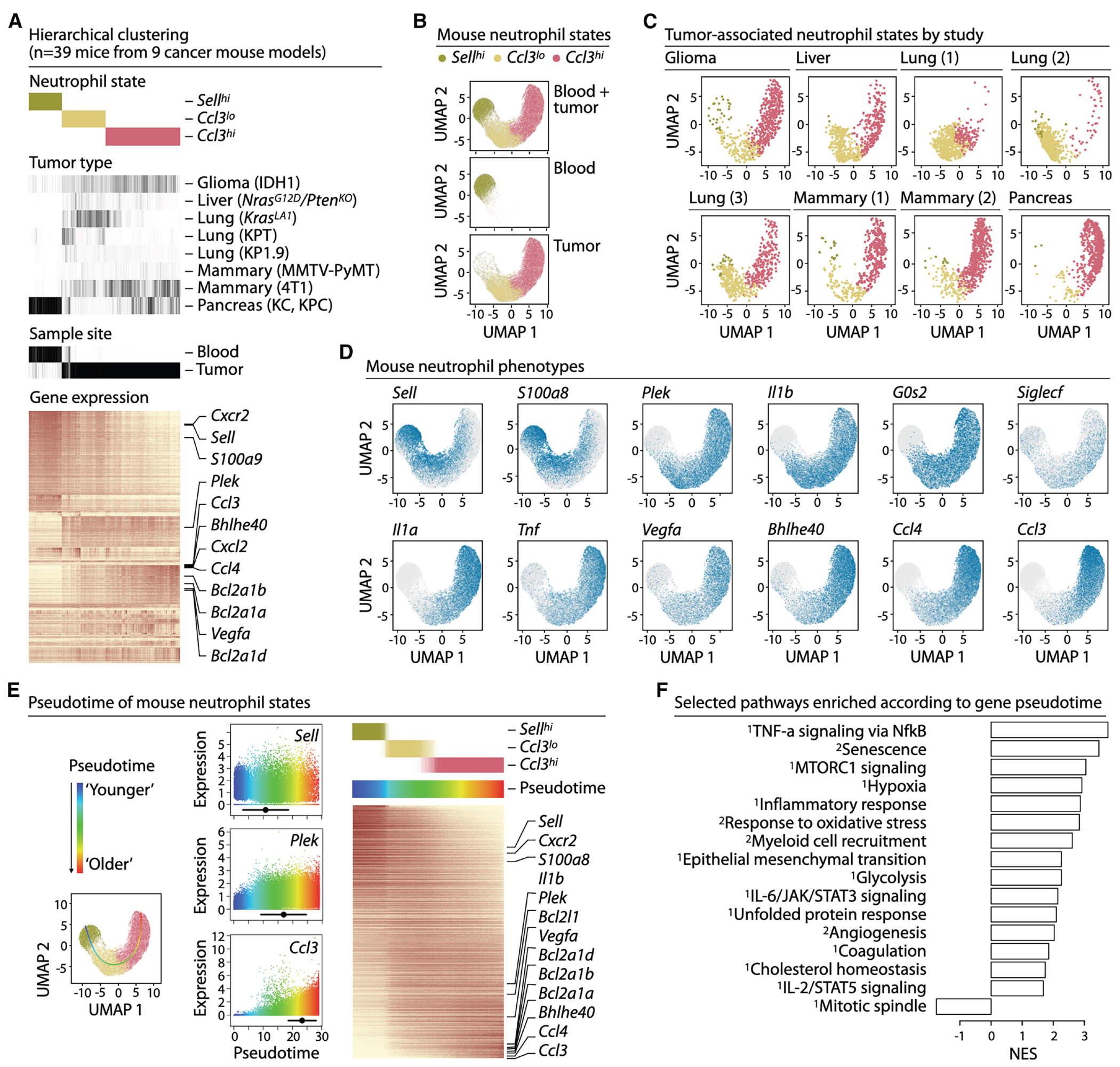
*Ccl3*^*hi*^ neutrophils mark an aged, pro-tumor state across mouse tumors (A) Hierarchical clustering of 8 mouse tumor scRNAseq datasets^30,56–62^ (*n* = 39 mice, Table S2) filtered by cell type probability, including blood and tumor samples. Three primary neutrophil states were identified: a blood-like *Sell*^*hi*^, *Ccl3*^*lo*^, and *Ccl3*^*hi*^ TAN. (B and C) UMAPs of combined (upper), blood-only (middle), tumor-only (lower) (b), and per study (c) neutrophil heterogeneity. (D) UMAP expression pattern of some example genes. (E) Pseudotime computed using a principal curve from “young” (*Sell*^hi^) to the most terminal “old” state (*Ccl3*^*hi*^) (left). Expression-weighted mean pseudotime and its standard deviation are summarized for each gene. Representative genes shown: *Sell*, *Plek*, *Ccl3* (middle). Heatmap displaying pseudotime-ordered cells and genes (right). (F) Genes ordered by pseudotime were used in gene set enrichment analysis (GSEA) against MSigDB hallmark gene sets^52^ (identified with the sign “^1^”) and curated tumor neutrophil gene sets^12,26,53^ (identified with the sign “^2^”). NES; Normalized Enrichment Score.

Hierarchical clustering distinguished tumor and blood neutrophils and revealed shared TAN states across tumor models (Figure 3A). Mouse neutrophils formed a continuum of states that spectral clustering resolved into three main subsets: *Sell^hi^*, *Ccl3^lo^*, and *Ccl3^hi^* (Figures 3A–3C). *Sell^hi^* neutrophils, enriched in blood (Figure 3B), expressed naive-associated genes such as *Sell*, *Cxcr2*, and *S100a8* (Figures 3A, 3D, and S4A). *Ccl3^lo^* and *Ccl3^hi^* neutrophils were tumor-enriched and showed increased expression of *Plek, Il1b*, and *G0s2* (Figures 3A, 3D, and S4A). Among these, *Ccl3^hi^* neutrophils showed higher expression of pro-tumor genes such as *Ccl4*, *Vegfa*, and *Bhlhe40* (Figures 3A, 3D, and S4A), reflecting human neutrophil phenotypes.

Some *Ccl3^hi^* TANs expressed *Siglecf*, a marker of tumor-promoting neutrophils in lung cancer,^12,30,63,64^ but this enrichment was limited to certain tumor models (Figures S4B and S4C). Other reported pro-tumor markers – *Tnfrsf23* (DcTRAIL R1), *Olr1* (LOX-1), *Tfrc* (CD71), *Slc27a2* (FATP2), and *Cd300ld* – were inconsistently expressed across tumor models and at lower levels than *Ccl3* (Figure S4C), highlighting their context specificity.^7,14,17,20,37^ These results suggest that *Ccl3* is a more robust and broadly applicable TAN marker in mice than *Siglecf* and other proposed markers. Furthermore, whereas *Siglecf* and *Tnfrsf23* are mouse-specific, *Ccl3* is conserved across species, enabling direct comparison between human and murine TANs.

Gene expression patterns across mouse neutrophil states were gradual (Figures 3A, 3D, and S4A). Principal curve fitting revealed a pseudotime trajectory from *Sell^hi^* to *Ccl3^lo^* to *Ccl3^hi^* cells (Figures 3E and S4D), mirroring the human *SELL^hi^*→*CCL3^hi^* progression. CytoTrace2^54^ confirmed this ordering (Figure S4E). Gene set enrichment along pseudotime identified pathways associated with pro-tumor functions (e.g., senescence, hypoxia, and response to oxidative stress) as increasingly activated toward the *Ccl3^hi^* state (Figure 3F).

The three-state continuum also aligned with previously reported murine TAN subtypes^17,30^ (Figure S5A). For example, *Ccl3^hi^* TANs corresponded to pro-tumor N4/N5 lung neutrophils from Zilionis et al.^30^ and T3 pancreatic neutrophils from Ng et al.^17^ Context-specific programs also mapped within this framework. For example, ISG^+^ neutrophils^26^ mapped to our clusters and could appear at any part of the *Sell^hi^*→*Ccl3^hi^* differentiation trajectory. (Figure S5B).

Collectively, these results indicate that murine tumors harbor stereotyped neutrophil states that follow a gradual differentiation trajectory culminating in a terminal, pro-tumor *Ccl3^hi^* state, with additional context-dependent programs that can be layered onto this shared framework. CCL3 emerges as a robust and conserved marker of these terminal neutrophils in mice.

### *Ccl3*^+^ mouse neutrophils resemble *CCL3*^+^ human neutrophils

**Figure 4.**
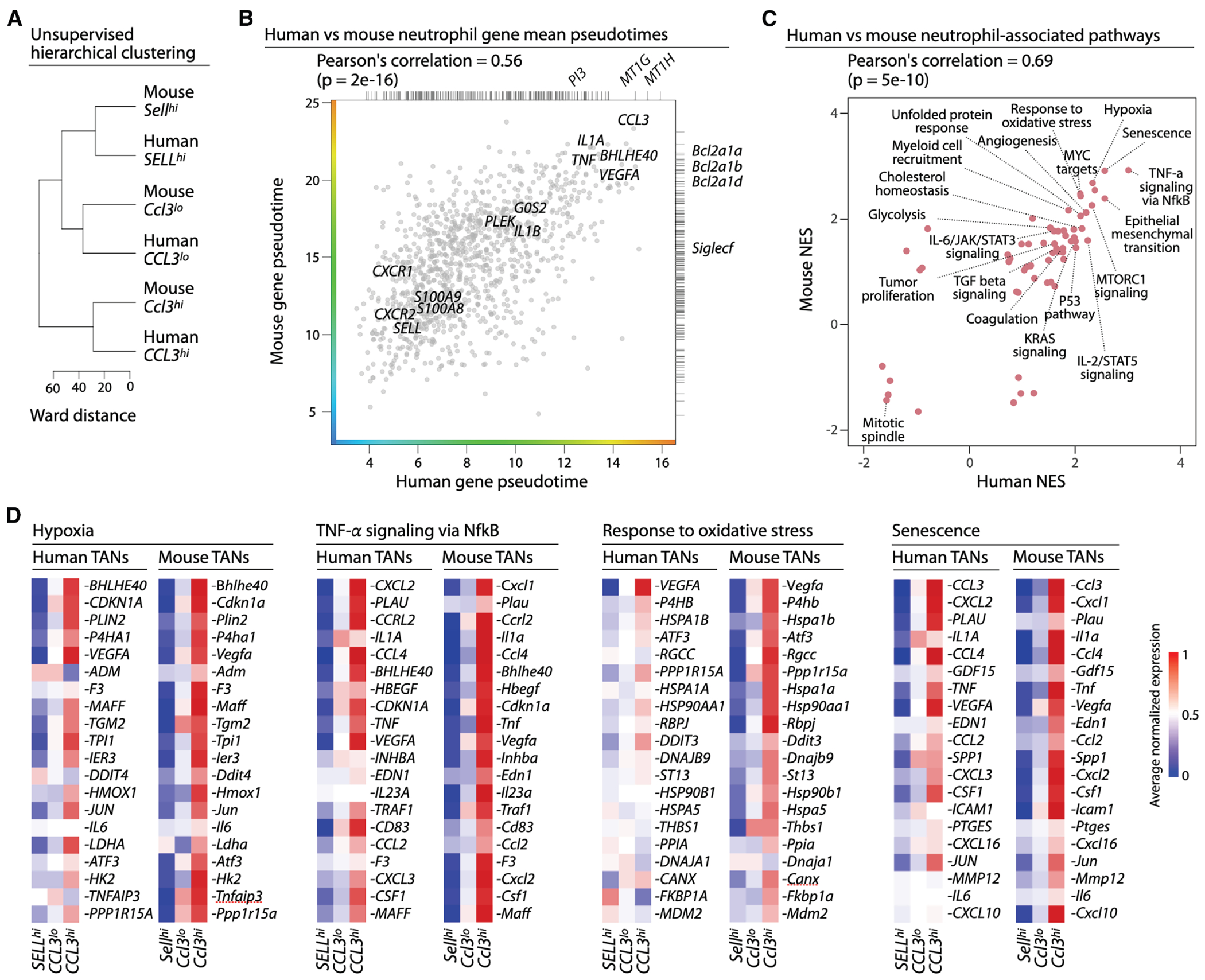
*Ccl3*^*hi*^ mouse neutrophils resemble *CCL3*^*hi*^ human neutrophils (A) Unsupervised hierarchical clustering of human and mouse neutrophil states based on average gene expression per neutrophil subtype. (B) Comparison of human and mouse neutrophil gene mean pseudotimes. Genes found in only one species are shown as ticks along the right and top margins. (C) Comparison of human and mouse normalized enrichment scores (NES) for all MSigDB hallmark and curated tumor neutrophil gene sets. (D) Heatmaps show average gene expression of top genes of pathways enriched in *CCL3*^*hi*^ neutrophils. Data for human and mouse neutrophil subsets are shown.

To compare neutrophil states in humans and mice, we used three complementary approaches. First, we performed unsupervised hierarchical clustering of neutrophil states from both species using the average expression of orthologous variable genes in these states. This analysis revealed a clear one-to-one correspondence between the three mouse and three human neutrophil states (Figure 4A).

Second, we compared gene progression along the human and mouse pseudotime trajectories. For each species, we calculated the mean pseudotime of orthologous genes and observed a strong correlation, indicating highly similar differentiation dynamics (Figure 4B). Genes at the positive extreme of this correlation – those increasing along pseudotime in both species – included *CCL3/Ccl3*, as well as pro-tumor genes such as *BHLHE40/Bhlhe40*, *VEGFA/Vegfa*, and IL1A/Il1a.^12,17,30,35,36,65^

Third, we compared normalized enrichment scores (NESs) across human and mouse gene sets. Gene set enrichment profiles were strongly correlated (Figure 4C). Pathways positively enriched in both species included hypoxia and senescence, whereas the negative extreme was dominated by the mitotic spindle. Orthologous gene sets associated with these pathways showed highly similar expression patterns in both mouse and human neutrophils (Figure 4D).

These analyses showed that neutrophil states and their transcriptional programs are conserved across cancers and between humans and mice, enabling consistent classification and cross-species comparison. The shared pro-tumor signatures, notably within the *CCL3^hi^*/*Ccl3^hi^* compartment, support the use of mouse models to study neutrophil-driven tumor progression. Among conserved features, *CCL3/Ccl3* emerged as one of the most robust markers of pro-tumor neutrophils, distinguishing it from previously proposed candidates,^7,12,14,17,20,37^ and motivating its mechanistic investigation in subsequent analyses.

### Neutrophil-derived CCL3 promotes tumor growth in mice

CCL3 is produced by multiple cell types (Figure S6A) and has diverse roles in tumor biology,^66–71^ but the contribution of neutrophil-derived CCL3 to tumor progression remains unclear. We addressed this question using two complementary genetic approaches to selectively manipulate *Ccl3* expression in mouse neutrophils.

**Figure 5.**
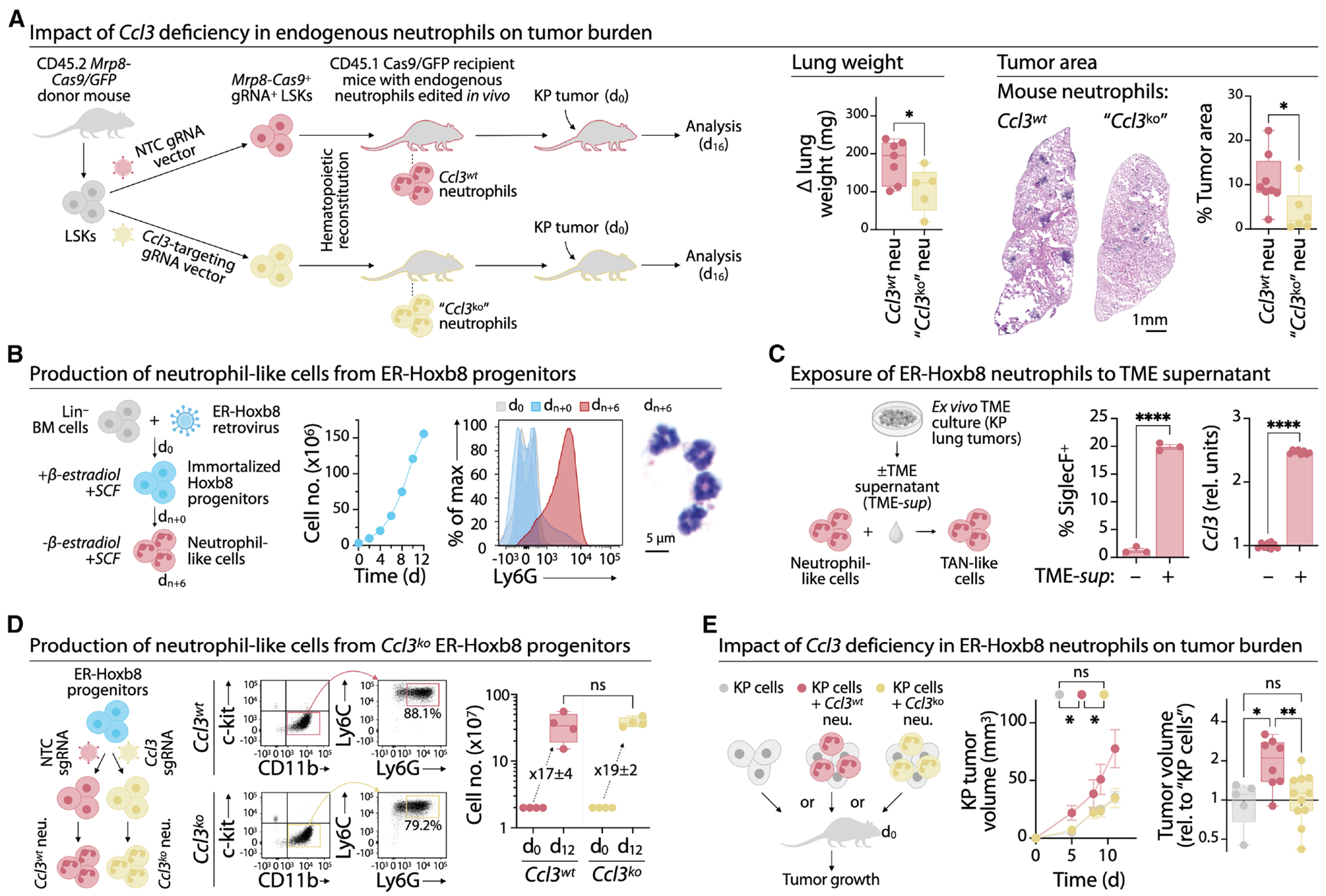
Neutrophil-derived CCL3 promotes tumor growth in mice (A) *In vivo* CRISPR/Cas9 editing schematic (left). KP1.9 cells were i.v. injected post-bone marrow reconstitution; at day 16, lung tumor burden was measured by lung weight as a proxy (normalized to non-tumor-bearing lungs- Δ lung weight, middle), and quantified on Hematoxylin & Eosin (H&E)-stained sections using QuPath v0.4.0 (right). n = 6–8 mice per group. Unpaired *t* test. (B) Schematic of ER-Hoxb8 neutrophil progenitor generation (left). Expansion of ER-Hoxb8 neutrophil progenitors in β-estradiol media quantified by hemacytometer (middle). Differentiation to CD45.1^+^ CD11b^+^ Ly6C^int^ Ly6G^+^ neutrophils after 6-day culture in differentiation medium, assessed by flow cytometry and Giemsa staining (right). (C) Schematic of the *in vitro* differentiation of ER-Hoxb8 neutrophils to TAN-like cells cultured for three days in control differentiation media or TME supernatant (TME-sup) (left). The proportion of SiglecF^+^ neutrophils was measured by flow cytometry at day 3 of culture (middle). *n* = 3 replicates per group; representative of multiple experiments. *Ccl3* expression in MACS-sorted Ly6G^+^ neutrophils at day 3 of culture was determined by qPCR. Expression levels are relative to cells cultured in control differentiation media (right). *n* = 8 replicates per group from 3 independent sorts. Unpaired *t* test. (D) Schematic of CRISPR/Cas9 editing in ER-Hoxb8 neutrophil progenitors (left). *Ccl3*^wt^ or *Ccl3*^ko^ ER-Hoxb8 neutrophil progenitor differentiation into mature CD45.1^+^ CD11b^+^ Ly6C^int^ Ly6G^+^ neutrophils in differentiation medium was evaluated by flow cytometry (middle), and their cell density in β-estradiol media was measured using a hemacytometer (right). (E) Schematic of co-injecting KP1.9 cells with *Ccl3*^wt^ or *Ccl3*^ko^ ER-Hoxb8 TANs subcutaneously (s.c.) into mice (left). Tumor volume was measured over 11 days (middle). two-way ANOVA. Day 8 tumor volume relative to KP1.9-control shown (right). Ordinary one-way ANOVA. n = 8–11 mice per group. KP1.9 cells alone served as a control. *n* = 5 mice. Box and whiskers plot (min to max) are presented in (a), (d), (e-right). Graphs in (c), (e-middle) show mean ± SEM. *p < 0.05, **p < 0.01, ****p < 0.0001.

First, we established a CRISPR/Cas9 strategy to delete *Ccl3* selectively in neutrophils *in vivo* (Figure 5A). Lin^−^ Sca1^+^ c-Kit^+^ cells (LSKs) were isolated from donor mice expressing neutrophil-specific *Cas9/GFP*, transduced with *Ccl3-*targeting or non-targeting (NTC) guide RNAs (gRNAs), and transferred into irradiated recipients for hematopoietic reconstitution. We compared Mrp8-Cas9/GFP and Ly6g-Cas9/GFP donors, finding that Mrp8-Cre drove robust, neutrophil-specific GFP expression (Figures S6B–S6D), consistent with prior studies.^14,20^ Thus, donor Mrp8-Cas9/GFP donors were used to generate recipients with *Ccl3*-deficient (“*Ccl3^ko^*”) or wild-type (“*Ccl3^wt^*”) neutrophils. Both groups showed comparable baseline characteristics after reconstitution, including body weight (Figure S6E) and immune composition (Figure S6F). Mice were then injected with KP1.9 cells, which form KRAS^G12D^/p53^null^ lung tumors^72^ associated with neutrophil recruitment,^73^ and analyzed 16 days later (Figure 5A). Mice with *Ccl3^ko^* neutrophils had significantly lower lung weights and reduced tumor areas than *Ccl3^wt^* controls, indicating that loss of *Ccl3* in endogenous neutrophils was sufficient to impair tumor growth *in vivo*.

Second, we developed a scalable strategy to generate large numbers of *Ccl3^ko^* neutrophils using the ER-Hoxb8 conditional myeloid differentiation model^43,44^ (Figures 5B and S7A). ER-Hoxb8 progenitors proliferate indefinitely in β-estradiol and differentiate synchronously into neutrophil-like cells upon hormone withdrawal. When exposed to KP1.9 lung tumor supernatant (TME-sup) for three days, ER-Hoxb8 neutrophils upregulated SiglecF and *Ccl3*, indicating the acquisition of TAN-like phenotypes^12,63,64^ (Figure 5C). *In vivo*, CD45.1 ER-Hoxb8 progenitors transferred into CD45.2 tumor-bearing mice gave rise to TANs resembling endogenous neutrophils (Figure S7B). These cells progressively transitioned from SiglecF^lo^ to SiglecF^hi^ TANs (Figure S7C), mirroring the transcriptomic “aging” trajectory (Figures 2E and 3E). A similar progression occurred *in vitro* (Figure S7D).

To engineer *Ccl3*^ko^ ER-Hoxb8 neutrophils, we derived progenitors from Cas9/GFP CD45.1 mice and transduced them with *Ccl3* or NTC gRNAs (Figure S7E). *Ccl3^ko^* and *Ccl3^wt^* progenitors produced comparable numbers of neutrophil-like cells (Figure 5D), indicating preserved viability and differentiation. To assess the impact of *Ccl3* in ER-Hoxb8 neutrophils on tumor burden *in vivo*, KP1.9 tumor cells were co-injected with either *Ccl3^ko^* or *Ccl3^wt^* cells into mice at a 1:4 ratio. Control mice received tumor cells alone. *Ccl3^wt^* neutrophils markedly increased tumor burden compared to controls, whereas *Ccl3^ko^* neutrophils failed to do so (Figure 5E). These results indicate that neutrophil-derived CCL3 promotes tumor growth in mice.

### Neutrophil-derived CCL3 sustains pro-tumor neutrophil survival

**Figure 6.**
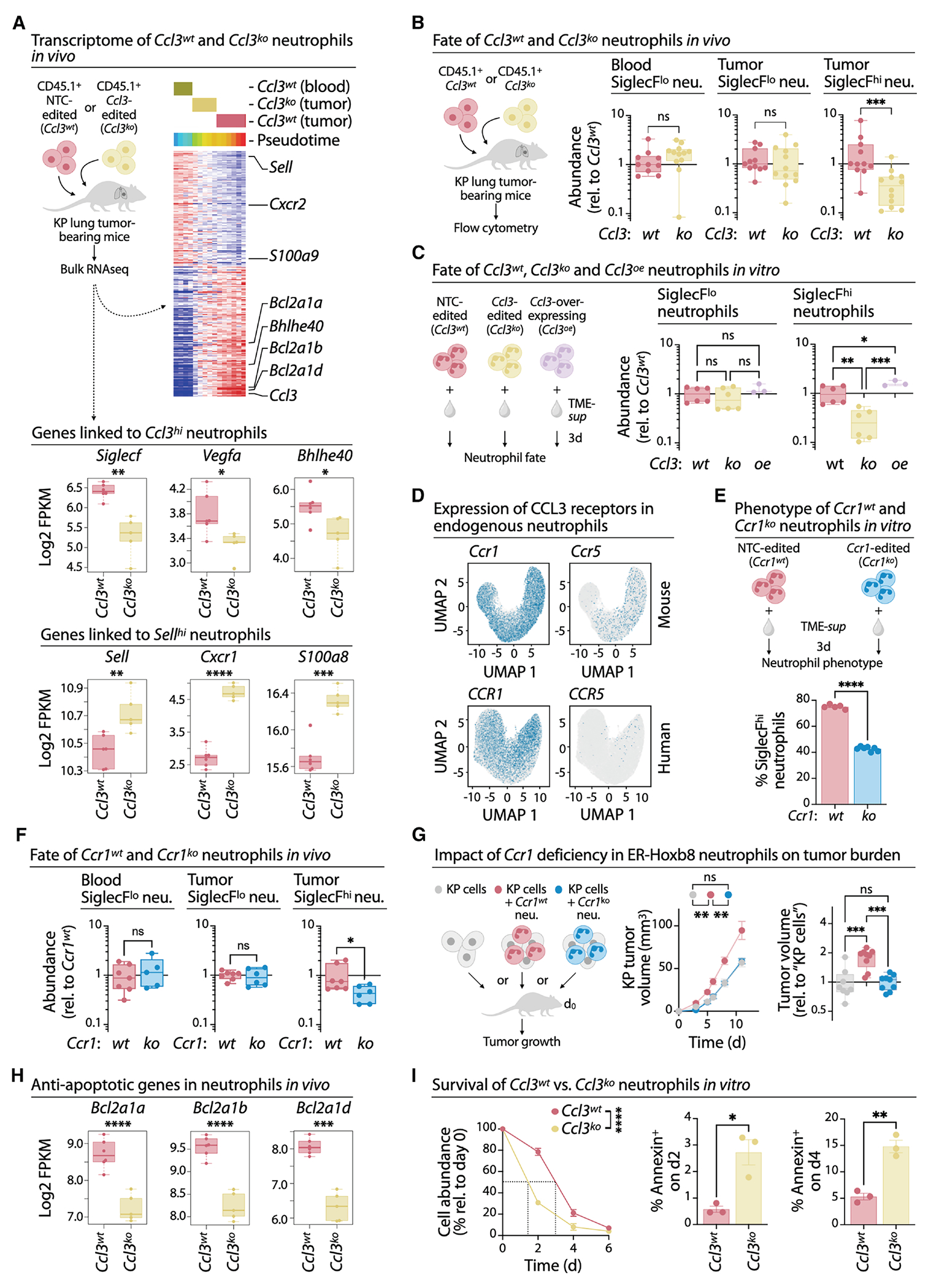
Neutrophil-derived CCL3 sustains pro-tumor neutrophil survival (A) Bulk RNAseq of *Ccl3*^*wt*^ or *Ccl3*^*ko*^ ER-Hoxb8 neutrophils isolated from the blood and tumor-bearing lungs of mice with orthotopic KP1.9 tumors by flow cytometry, as CD45.1^+^CD11b^+^Ly6G^+^ cells. Upper panel: Heatmap of average gene expression profiles derived from the pseudotime gene set (see Figure 3E) comparing blood *Ccl3*^*wt*^, tumor *Ccl3*^*wt*^, and tumor *Ccl3*^*ko*^ ER-Hoxb8 neutrophils. Lower panel: Log expression of selected genes in tumor *Ccl3*^*wt*^ and *Ccl3*^*ko*^ ER-Hoxb8 neutrophils. n = 5–6 mice per group. Wilcoxon test. (B) Relative abundance of SiglecF^lo^ and SiglecF^hi^ *Ccl3*^wt^ or *Ccl3*^*ko*^ ER-Hoxb8 neutrophils in blood and KP1.9 tumor-bearing lungs, measured by flow cytometry. Abundance refers to absolute cell counts per mg lung tissue, relative to median *Ccl3*^wt^ counts. *n* = 10–12 mice per group, pooled from 2 independent experiments. Unpaired *t* test. (C) Relative abundance of SiglecF^lo^ and SiglecF^hi^ cells in *Ccl3*^wt^, *Ccl3*^*ko*^, or *Ccl3*^*oe*^ ER-Hoxb8 TANs *in vitro*, cultured for three days in TME-sup and measured by flow cytometry at day 3. Abundance refers to absolute cell counts relative to average *Ccl3*^wt^ counts. n = 3–6 replicates per group. Ordinary one-way ANOVA. (D) UMAP visualization of *Ccr1/CCR1* and *Ccr5/CCR5* expression in mouse and human TANs across multiple cancers (dataset shown in Figures 2 and 3; Tables S1 and S2). (E) Proportion of SiglecF^hi^ in *Ccr1*^wt^ or *Ccr1*^*ko*^ ER-Hoxb8 TANs *in vitro*, cultured for three days in TME-sup and measured by flow cytometry at day 3. n = 5–7 replicates per group. Unpaired *t* test. (F) Relative abundance of SiglecF^lo^ and SiglecF^hi^ *Ccr1*^wt^ or *Ccr1*^*ko*^ ER-Hoxb8 neutrophils in blood (left) and KP1.9 tumor-bearing lungs (middle-right), measured by flow cytometry. Abundance refers to absolute cell counts per mg lung tissue, relative to median *Ccr1*^wt^ counts. n = 5–7 mice per group. Unpaired *t* test. (G) Schematic of co-injecting KP1.9 cells with *Ccr1*^*wt*^ or *Ccr1*^*ko*^ ER-Hoxb8 neutrophils s.c. into mice (left). Tumor volume was measured over 11 days (middle). two-way ANOVA. Day 8 tumor volume relative to KP1.9-control shown (right). Ordinary one-way ANOVA. n = 8–9 mice per group. (H) Log expression of anti-apoptotic *Bcl2* family genes from bulk RNA-seq in *Ccl3*^wt^ and *Ccl3*^ko^ ER-Hoxb8 TANs isolated from orthotopic KP1.9 lung tumors. n = 5–6 mice per group. Wilcoxon test. (I) Fold change in *Ccl3*^*wt*^ or *Ccl3*^*ko*^ ER-Hoxb8 TAN abundance relative to day 0 after three-day culture in TME-sup for TAN differentiation, followed by flow cytometric analysis at days 2, 4, and 6 to assess survival (left). Abundance refers to absolute cell counts. *n* = 3 replicates per group. two-way ANOVA. Apoptosis measured by Annexin V staining and flow cytometry (right). *n* = 3 replicates per group. Representative of two independent experiments. Unpaired *t* test. Box and whiskers plot (min to max) are presented in (a), (b), (c), (f), (g-right), (h). Graphs in (e), (g-middle), (i-right) show mean ± SEM, and in (i-left) show mean ± SD. *p < 0.05, **p < 0.01, ***p < 0.001, ****p < 0.0001, ns, non-significant.

We next tested how neutrophil-derived CCL3 supports TAN protumor functions using the ER-Hoxb8 system. CD45.1 *Ccl3*^wt^ or *Ccl3*^ko^ ER-Hoxb8 progenitors were transferred intravenously into CD45.2 mice bearing orthotopic KP1.9 lung tumors, and CD45.1 TANs were isolated five days later for bulk RNAseq. *Ccl3^ko^* TANs showed reduced expression of genes associated with aged or terminal cells (*Siglecf*, *Vegfa*, *Bhlhe40*) and increased expression of genes associated with younger cells (*Sell*, *Cxcr1*, *S100a8*) (Figure 6A). Pseudotime ordering likewise placed *Ccl3^ko^* neutrophils closer to less differentiated states (Figures 6A and S8A).

To assess whether *Ccl3* affects general neutrophil development or specifically terminal differentiation, we measured the capacity of CD45.1 *Ccl3^wt^* and *Ccl3^ko^* progenitors to produce SiglecF^lo^ and SiglecF^hi^ neutrophils in CD45.2 KP1.9 tumor-bearing mice (Figure 6B). In this model, SiglecF marks aged, pro-tumor neutrophils.^12,63,64^ In blood, all CD45.1 neutrophils remained SiglecF^lo^ as expected,^12,63^ with *Ccl3^ko^* and *Ccl3*^wt^ neutrophils present in similar numbers. In tumors of comparable burden (Figure S8B), SiglecF^lo^ *Ccl3^ko^* neutrophils also accumulated at levels comparable to *Ccl3^wt^* cells, but SiglecF^hi^ *Ccl3^ko^* neutrophils were reduced (Figure 6B). Thus, CCL3 is dispensable for neutrophil development, infiltration, or early maturation but is necessary for the accumulation of terminally differentiated TANs.

We modeled these dynamics *in vitro* by exposing *Ccl3^wt^* and *Ccl3^ko^* ER-Hoxb8 cells to TME-sup. Both genotypes upregulated SiglecF after one day (Figure S8C). After three days, the abundance of SiglecF^lo^ neutrophils remained similar, but *Ccl3^ko^* markedly reduced the proportion of SiglecF^hi^ cells (Figures 6C and S8D), a result replicated with a second *Ccl3*-targeting guide (Figure S8E). Conversely, *Ccl3*-overexpressing (*Ccl3^oe^*) neutrophils produced more SiglecF^hi^ cells (Figure 6C). These data indicate that CCL3 promotes terminal TAN accumulation rather than early differentiation.

Because these experiments used isolated neutrophils, we hypothesized that these cells can both produce and sense CCL3. Among CCL3 receptors, only CCR1 – not CCR4 or CCR5 – was robustly expressed in human and murine neutrophils at both gene and protein levels (Figures 6D, S8F, and S8G). To test CCR1 function, we generated *Ccr1*-deficient ER-Hoxb8 progenitors and examined neutrophil phenotypes after three days of TME-sup exposure. *Ccr1^ko^* neutrophils showed reduced numbers of SiglecF^hi^ cells compared to *Ccr1^wt^* controls (Figures 6E and S8H). *In vivo*, *Ccr1^ko^* progenitors produced normal SiglecF^lo^ neutrophil counts but significantly fewer SiglecF^hi^ TANs (Figure 6F). Functionally, *Ccr1^wt^* neutrophils promoted KP1.9 tumor growth, whereas *Ccr1^ko^* TANs did not (Figure 6G), mirroring the effects of *Ccl3* loss and identifying a CCL3-CCR1 survival axis.

Transcriptomic profiling showed that *Ccl3*^ko^ TANs had reduced expression of anti-apoptotic *Bcl2* family genes *in vivo* (Figure 6H), which are linked to the extended survival of SiglecF^hi^ lung neutrophils.^64^ This pattern was recapitulated *in vitro* (Figure S8I). Consistent with this, treating *Ccl3*^wt^ neutrophils with pro-apoptotic inhibitors of BCL2-family proteins (BH3 mimetics^64^) reduced their survival to levels resembling those of *Ccl3^ko^* cells (Figure S8J).

Survival assays confirmed this mechanism: *Ccl3*^ko^ neutrophils declined faster in TME-sup cultures than *Ccl3*^wt^ cells (Figure 6I). After two days, 79 ± 3% of *Ccl3*^wt^ neutrophils remained versus only 31 ± 2% of *Ccl3*^ko^ neutrophils; half-lives were ~3 and ~1.5 days, respectively. Annexin V staining on days 2 and 4 showed more apoptotic cells among *Ccl3*^ko^ neutrophils (Figure 6I). Thus, neutrophil-derived CCL3 supports the survival of pro-tumor neutrophils.

### Hypoxic tumor niches promote the acquisition of the CCL3^hi^ neutrophil state

**Figure 7.**
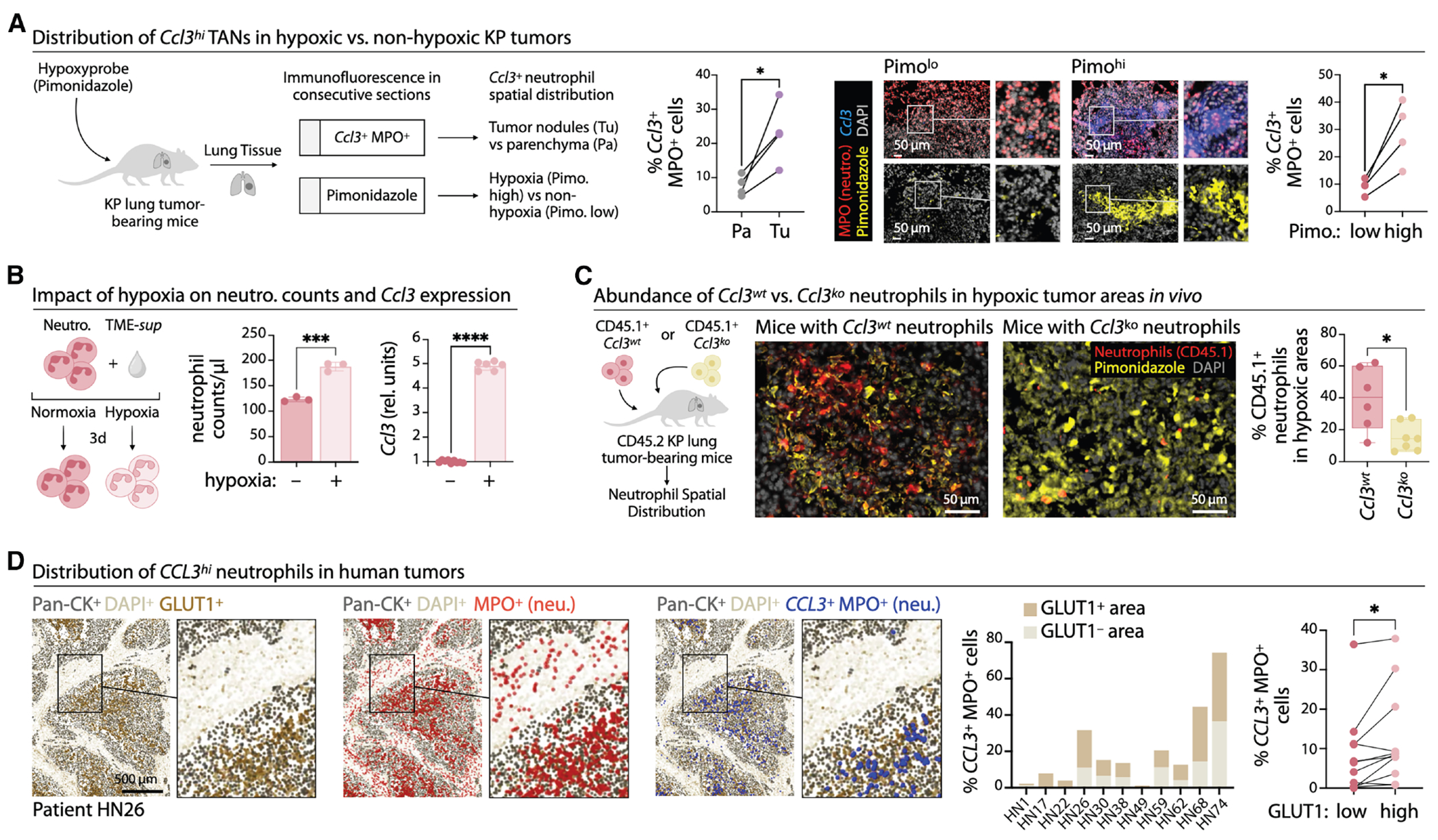
Hypoxic tumor niches promote the acquisition of the CCL3^hi^ neutrophil state (A) Workflow schematic (left). Proportion of *Ccl3*^*hi*^ MPO^+^ neutrophils normalized to total MPO^+^ cells in tumor nodules (Tu) versus lung parenchyma (Pa) (middle) and pimo^lo^ versus pimo^hi^ areas (right), measured by immunofluorescence staining or RNAscope in consecutive sections. Representative images shown. *n* = 4 mice. Paired *t* test. (B) Schematic of ER-Hoxb8 neutrophil differentiation into TAN-like cells in TME-sup under hypoxia or normoxia (left). ER-Hoxb8 TAN counts measured by flow cytometry at day 3 (middle). *n* = 3 replicates per group. Representative of two independent experiments. *Ccl3* expression in MACS-sorted TANs measured by qPCR. Expression levels are shown relative to normoxia (right). *n* = 6 replicates per group from 2 independent sorts. Unpaired *t* test. (C) Schematic of the spatial distribution of *Ccl3*^wt^ or *Ccl3*^ko^ ER-Hoxb8 TANs in hypoxic areas *in vivo* five days post-transfer (left). Representative immunofluorescence images: ER-Hoxb8 TANs identified by CD45.1 expression, and hypoxic regions marked by pimonidazole staining (middle). Proportion of CD45.1^+^ *Ccl3*^wt^ or *Ccl3*^ko^ ER-Hoxb8 neutrophils in hypoxic areas measured by immunofluorescence staining (right). n = 6–7 mice per group. Unpaired *t* test. (D) Distribution of *CCL3*^*hi*^ MPO^+^ neutrophils in patients with HNSCC. Left panels: Representative images in GLUT1^hi^ versus GLUT1^lo^ areas. Right panels: Proportion normalized to total MPO^+^ cells in GLUT1^hi^ versus GLUT1^lo^ Pan-CK^+^ areas, as assessed by RNAscope. *n* = 11 patients. Paired *t* test. Box and whiskers plot (min to max) are presented in (c). Bar graphs in (b) show mean ± SEM. *p < 0.05, ***p < 0.001, ****p < 0.0001.

Given that hypoxia was one of the most strongly enriched pathways in *Ccl3^hi^*/*CCL3^hi^* TANs across mouse and human cancers (Figures 4C and 4D), we asked whether hypoxia regulates *Ccl3* expression. We mapped the spatial distribution of mouse *Ccl3^hi^* TANs in orthotopic KP1.9 lung tumors using pimonidazole to label hypoxic regions (Figure 7A). *Ccl3^hi^* TANs localized mainly within tumor nodules and were enriched in hypoxic (pimo^hi^) regions, whereas *Ccl3^−/lo^* TANs were equally found in pimo^lo^ and pimo^hi^ regions (Figure S9A). Thus, while TANs may enter hypoxic zones independently of CCL3, CCL3 expression is associated with hypoxia exposure.

To test whether hypoxia influences TANs, we cultured ER-Hoxb8 neutrophils in TME-sup under hypoxic or normoxic conditions. After 24 h, cell abundance was similar (Figure S9B), indicating that short-term survival was oxygen-independent. By day three, however, hypoxia increased neutrophil abundance and strongly induced *Ccl3* (Figures 7B, S9B, and S9C). This survival advantage was lost in *Ccl3^ko^* neutrophils (Figure S9D). In hypoxia-survival assays, *Ccl3^ko^* neutrophils declined faster than *Ccl3^wt^* (Figure S9E), with selective loss of aged SiglecF^hi^ cells but preservation of SiglecF^lo^ cells (Figure S9F). Thus, prolonged hypoxia enhances TAN survival in a CCL3-dependent manner, particularly within the terminal SiglecF^hi^ population.

To validate these findings *in vivo*, CD45.1 *Ccl3^wt^* or *Ccl3^ko^* ER- Hoxb8 progenitors were transferred into CD45.2 KP1.9 lung tumor-bearing mice with comparable tumor burden (Figure S9G). *Ccl3^ko^* TANs were underrepresented in hypoxic regions (Figure 7C), showed reduced pimonidazole staining (Figure S9H), and exhibited lower expression of hypoxia-inducible genes (Figure S9I). Thus, while TANs can access hypoxic niches without CCL3, their persistence and adaptation within these niches require it. To extend these observations to patients, we analyzed tumor sections from eleven HNSCC cases. *CCL3^hi^* TANs were enriched in hypoxic, glycolytic Pan-CK^+^ tumor regions marked by high GLUT1 expression^45^ (Figure 7D), mirroring the murine findings. These data indicate that hypoxia induces CCL3 expression in TANs and supports a CCL3-dependent survival program, enabling the accumulation and activity of *CCL3^hi^* pro-tumor neutrophils within hypoxic tumor niches.

## DISCUSSION

This study establishes a unified framework for analyzing TANs, revealing conserved neutrophil states across cancers and identifying CCL3 as a central regulator of neutrophil-driven tumor progression.

A contribution of this work is the development of a tailored classifier that resolves the long-standing underrepresentation of neutrophils in scRNAseq datasets. Conventional pipelines remove many neutrophils because they apply uniform quality-control thresholds that fail to account for the cells’ inherently low mRNA content, while existing reference-based tools cannot reliably identify neutrophils in unfiltered matrices containing large numbers of debris-associated barcodes. By integrating cell-type prediction and quality assessment within a single probability model, our approach recovers neutrophils with high fidelity across diverse datasets. Optimized protocols exist to enhance neutrophil capture^25,32,35^; however, they cannot be retroactively applied, whereas our method enables reanalysis of existing studies, provided unfiltered barcodelevel data are available. Importantly, TAN abundance varied markedly across tumors, correlated with protein-based measurements and clinical outcomes, and therefore represents meaningful biological heterogeneity rather than technical noise.

Using this improved detection framework, we found that neutrophils form a continuous differentiation trajectory from circulating young *SELL^hi^/Sell^hi^* cells to terminal old *CCL3^hi^/ Ccl3^hi^* cells enriched in tumors. This continuum aligns with previous observations^30^ and with epigenetic maturation processes reported in pancreatic cancer.^17^ Our results extend these concepts across tumor types, individuals, and species, indicating that they reflect shared biological principles rather than cohort- or tissue-specific artifacts, and providing a coherent model of neutrophil differentiation in cancer. Although our analysis is compatible with previously reported context-specific neutrophil programs,^26,55^ it is designed to identify conserved neutrophil states. Local microenvironmental factors, including cancer- and tissue-specific cues, can impose additional transcriptional programs, and defining these context-dependent adaptations represents an important direction for future investigation.

Several markers, including SiglecF in KP lung tumors,^12^ dcTRAIL-R1 in KPC pancreatic tumors,^17^ and CD71 in brain tumors,^37^ have been individually associated with pro-tumor, long-lived TANs. However, these markers varied across cancers and species. In contrast, *CCL3*/*Ccl3* was consistently elevated in the terminal TAN state and aligned strongly with previously described pro-tumor subsets, such as SiglecF^hi^ and “T3” neutrophils^12,17^ in lung and pancreatic mouse tumors, respectively. These findings suggest that independently described populations across studies converge on a shared CCL3^hi^ program.

*CCL3^hi^/Ccl3^hi^* TANs were enriched in hypoxic tumor regions, where hypoxia can reinforce their terminal differentiation and pro-tumor function; similarly to the hypoxia-associated “T3” neutrophils previously described.^17^ These cells also exhibited a senescence-associated transcriptional signature, echoing findings that long-lived, senescent neutrophils promote tumor growth in prostate cancer.^74^ Clinically, *CCL3^hi^* TANs have been observed in anti-PD1-resistant NSCLC,^36^ underscoring their pathological relevance. Together, these findings identify *CCL3*^hi^ TANs as a conserved, functionally important pro-tumor neutrophil population.

Efforts to study TAN function have historically relied on global neutrophil depletion^2,34,41,42^ whereas tools for gene-specific perturbation in neutrophils remain limited. Recent platforms such as CHIME, a CRISPR-Cas9 chimera-based *in vivo* screening system,^75^ enable broad gene discovery but cannot pinpoint the cell of origin for genes of interest, underscoring the need for neutrophil-specific editing strategies. We therefore developed the *Mrp8-Cre-eGFP* × *Rosa26-LSL-Cas9-eGFP* model to selectively manipulate genes in neutrophils. Using this system, we found that *Ccl3* deletion in neutrophils reduces their survival and impairs tumor growth, establishing that neutrophil-derived CCL3 is a functional driver of tumor progression. Although CCL3 has been linked to TAN recruitment,^68–71^ macrophage-mediated metastasis,^67^ and immunosuppressive myeloid cell accumulation,^34^ its role in sustaining TAN survival had not been defined. Previous pharmacological targeting of CCL3- CCR1/5 could not distinguish cellular sources of CCL3 or separate its effects from those of related chemokines such as CCL4 or CCL5.^76^ Our data show that neutrophil-derived CCL3 maintains pro-tumor TANs in tumors.

Mechanistically, ER-Hoxb8-based perturbations revealed that *Ccl3^ko^* neutrophils have reduced survival, particularly within the long-lived SiglecF^hi^ subset. Hypoxia induced CCL3 expression and enhanced TAN survival, establishing a feedforward loop in which hypoxia promotes CCL3 expression, and CCL3 sustains TANs within hypoxic niches. Deleting CCR1, the primary CCL3 receptor on neutrophils, reduced SiglecF^hi^ neutrophil survival and abrogated their pro-tumor activity, indicating that CCL3- CCR1 signaling supports the persistence of terminal TANs. This suggests that neutrophils can both produce and sense CCL3, enabling an autocrine or locally amplifying circuit that stabilizes CCL3^hi^ neutrophils in specific microenvironmental niches, such as hypoxic areas. Defining the downstream survival pathways and the contributions of CCL3 from other cell types will be important future steps.

Our findings offer several avenues for clinical translation. First, the conserved nature of CCL3^hi^ TANs across patients and tumor types suggests their potential as a pan-cancer biomarker for prognosis and therapeutic response. Second, CCL3 represents a candidate molecular target for modulating neutrophil-driven tumor inflammation, particularly in hypoxic, therapy-resistant tumor regions. Defining the pro-survival pathways that sustain CCL3^hi^ TANs and understanding interactions with CCR1/ CCR4/CCR5^+^ immune or stromal cells may reveal spatially organized immunosuppressive niches. Third, the combination of high-resolution TAN mapping and neutrophil-specific gene editing strategies paves the way for identifying additional regulators of neutrophil differentiation and function, ultimately informing next-generation myeloid-targeted immunotherapies.

## RESOURCE AVAILABILITY

### Lead contact

Further information or requests for resources or reagents should be directed to and will be fulfilled by the lead contact, Prof. Mikaël J. Pittet (mikael.pittet@ unige.ch).

### Materials availability

All unique/stable reagents generated in this study are available from the lead contact with a completed materials transfer agreement.

## STAR★METHODS

### EXPERIMENTAL MODEL AND STUDY PARTICIPANT DETAILS

#### Animals

*Kras^LSL-G12D/WT^;p53^Flox/Flox^* (referred to as KP1.9) mice were used as a conditional mouse model of NSCLC^80^ and bred in our laboratory in the Gt(ROSA)26Sortm1.1(CAG-cas9*,-EGFP)Fezh/J (JAX 024858) (referred to as Cas9/GFP) or Gt(ROSA)26Sortm1.1(CAG-cas9*,-EGFP)Fezh/J CD45.1 STEM background (referred to as Cas9/GFP CD45.1). Gt(ROSA)26Sortm1.1(CAG-cas9*,-EGFP)Fezh/J CD45.1 STEM mice were generated at Simches Research Center (MGH, Boston) by crossing commercial Gt(ROSA)26Sortm1.1 (CAG-cas9*,-EGFP)Fezh/J mice (JAX 024858) with CD45.1-STEM (C57BL/6-CD45.1^STEM^) mice.^81^ Breeding of homozygotes Gt(ROSA)26Sortm1.1(CAG-cas9*,-EGFP)Fezh/J CD45.1 STEM was maintained in our laboratory (Agora *In Vivo* Center). Bone marrow chimera experiments were performed using hemizygotes *Mrp8-Cre-eGFP* x hemi or homozygotes *Rosa26-LSL-Cas9-eGFP* donors (referred to as *Mrp8-Cas9/GFP*) that were generated in our laboratory (Agora *In Vivo* Center) by crossing *B6.Cg-Tg(Mrp8-cre,-EGFP)1Ilw/J* (JAX 021614) with B6J.129(B6N)-Gt(ROSA)26Sortm1(CAG-cas9*,-EGFP)Fezh/J (JAX026175) (both purchased from Jackson Laboratory) or by crossing hemizygotes *Mrp8-Cas9/GFP* (hemizygotes *Mrp8-Cre-eGFP* x hemizygotes *Rosa26-LSL-Cas9-eGFP*). *Ly6G-Cas9/GFP* mice were generated at Simches Research Center (MGH, Boston) by crossing catchup *Ly6g-Cre mice* (provided by prof. Matthias Gunzer) with B6J.129(B6N)-Gt(ROSA)26Sortm1(CAG-cas9*,-EGFP)Fezh/J (JAX026175) (purchased from Jackson Laboratory). C57BL6/J (Cat #000664) were purchased from Jackson Laboratory. Upon arrival, mice purchased from Jackson were housed in our facility for at least one week before the start of the experiment. All animal experiments were approved by the Veterinary Authority of the Canton de Vaud and Genè ve, Switzerland (license number VD3612), the Ré seau des animaleries lé maniques (RESAL) competent ethic committee and the IACUC guidelines.

#### Tumor cell line

The KP1.9 cell line, derived from lung tumor nodules of a male C57BL/6 KP1.9 mouse, was maintained in Iscove’s DMEM media supplemented with 10% fetal bovine serum (FBS) and 1% penicillin/streptomycin as described previously.^12,72^

#### Human samples

Fresh tumor samples for TotalSeq and FFPE sections for histological analyses were collected from HNSCC patients with signed informed consent under IRB approved protocols [Massachusetts General Hospital (MGH) (IRB#2014P000559; IRB#13-416) and Massachusetts Eye and Ear (MEE) (IRB#11-024H)]. Clinical demographic information, such as age, sex, smoking, and others, were extracted from electronic medical records. Non-smokers were defined as having a smoking history of <10 pack years.

### METHOD DETAILS

#### Mouse studies

##### Tumor model

The KP1.9 cell line, derived from lung tumor nodules of a male C57BL/6 KP1.9 mouse, was maintained in Iscove’s DMEM media supplemented with 10% fetal bovine serum (FBS) and 1% penicillin/streptomycin as described previously.^12,72^ KP1.9 lung tumors were induced by intravenous (i.v.) tail vein injection of 2.5 x 10^5^ KP1.9 cells into male C57BL6/J mice, as described previously.^12,72^ Tumors were analyzed between 16-20 days after tumor induction.

#### Organ digestion

##### Lungs and KP1.9-bearing lungs

Were isolated and minced using surgical scissors, then digested with 1 mg/ml Collagenase I (Worthington) in RPMI-1640 at 37-degrees C for 30 minutes shaking at 900 rpm. Digested tumors were then processed through a 40 μm cell strainer, centrifuged at 1500 RPM for 5 minutes and subject to RBC lysis buffer for 1 minute at RT. After quenching with RPMI-1640 and centrifuging at 1500 RPM for 5 minutes, all samples were resuspended in 0.5% BSA in PBS for flow cytometry staining.

##### Bones (femur, tibia, arms)

Were harvested, and bone marrow was isolated following a published protocol.^82^ Harvested cells were processed through a 40 μm cell strainer and subject either to RBC lysis buffer for up to 5 minutes and resuspended in 0.5% BSA for flow cytometry staining or to Ficoll gradient and centrifuged at 800g, 20°C for 20 minutes with no breaks. Then, the interphase was collected for bone marrow reconstitution.

##### Blood

Was collected in 1.5 ml tubes with 20 μL of 50 mM EDTA to prevent clotting and subject to RBC lysis buffer. After quenching with RPMI-1640 and centrifuged for 5 minutes at 1500 RPM, cells were resuspended in 0.5% BSA in PBS for flow cytometry staining.

#### *In vivo* gene-editing of endogenous neutrophils via CRISPR/Cas9

Bone marrow cells were isolated from bones of Mrp8-Cas9/GFP (CD45.2) donors following the protocol described in “Organ digestion”. The isolated interphase of the Ficoll gradient was used for sorting of lineage negative cKit^+^ Sca1^+^ hematopoietic stem cells and progenitors (LSKs). First, cKit^+^ cells were enriched using MACS selection with anti-cKit magnetic beads following the provider’s protocol (Miltenyi Biotec, #130-091-224). Then, LSKs were isolated by FACS to ensure purity, using the following staining cocktail: lineage negative B220/CD45R^−^, CD19^−^, Ter119^−^, CD11c^−^, CD11b^−^, NK1.1^−^, Dx5/CD49b^−^, CD127^−^, Ly6G^−^, CD90.2^−^PE; c-kit^+^-APC; Sca-1^+^-BV421 (Abs are listed in key resources table). Sorted LSKs were cultured in 1ml StemSpan SFEM medium (STEMCELL Technologies, #09650) with a mixture of cytokines (100 ng/ml of recombinant mouse Flt3L #250-31L, IL-7 #217-17, SCF #250-03, TPO #315-14 from PeproTech, Rocky Hill, NJ) overnight in 24-well low-adherence plates (Corning, #3473) at 30,000 cells/ml.

The next day, LSKs were transferred into 6-well low-adherence plates (Corning, #3471), transduced with Non-Targeting Control (NTC) sgRNA or *Ccl3* sgRNA lentiviral vectors (detailed in Lentiviral library) and spiked with polybrene (8 μg/ml, Merck Millipore, #TR-1003-G) for spin-infection (1000g, 1hr at RT). LSKs were then diluted in StemSpan SFEM medium plus cytokines at 2 μg/ml polybrene and allowed to infect for two days.

After 2 days, successfully transduced NTC or *Ccl3* sgRNA^+^ LSKs were selected by FACS based on human CD19^+^ expression. Up to 160,000 cells were injected i.v. in Cas9/GFP CD45.1 recipient mice that had previously received a myeloablative dose of irradiation (2 x 4.5Gy) for bone marrow reconstitution using an X-Ray X-Strahl irradiator. After bone marrow transplantation, mice were placed into autoclaved cages for eight weeks and treated as immune-deficient animals for two weeks. At week 8 post-transplantation, the reconstituted mice were used for KP1.9 tumor growth experiments. In *Ccl3* sgRNA^+^ mice, *in vivo* activation of the *Mrp8* promoter aims to induce Cas9 expression specifically in neutrophils and to subsequently edit *Ccl3*. The efficiency of editing was determined by Next Generation Sequencing (NGS).

#### *In vitro* ER-Hoxb8 cell cultures

##### ER-Hoxb8 cell generation

ER-Hoxb8 cells derived from bone marrow cells of male C57BL/6J or Cas9/GFP CD45.1 STEM mice. Detailed protocols for construction of the retroviral vector encoding ER–Hoxb8, virus production, bone marrow cell preparation and transduction were described previously^43^ and are available online: https://www.hematopia.com/er-hoxb8. In brief, bone marrow mononuclear cells were isolated and expanded in medium containing recombinant mouse SCF, IL-6 (#78052.1 STEMCELL), and IL-3 (10ng/ml) (#213-13, Peprotech) for 48 hours. Cells were placed in 12-well plates pre-coated with human fibronectin (Sigma-Aldrich #F-0895) and spin-infected with murine stem cell virus vectors (MSCVneo) encoding ER–Hoxb8. Infected cells were cultured in RPMI-1640 medium containing recombinant mouse SCF (100 ng/mL) and β-estradiol (500 nM, Sigma-Aldrich, #50-28-2) for two days prior to addition of G418 (Gibco, #10131035) for selection. The cells were passaged to new plates every 3-4 days with fresh conditioned medium (RPMI-GlutaMax (Gibco, #61870-010), 10% FBS, 1% Pen/Strep, 1% SCF media (derived from a CHO-SCF cell line, equivalent to ~50-100 ng/ml), β-estradiol (0.5-M)). Non-adherent immortalized cells grew out in about 3 weeks. Stocks of ER-Hoxb8 cells were generated and cultured *in vitro* for 1 week prior to experiments using conditioned medium (RPMI-GlutaMax, 10% FBS, 1% Pen/Strep, 1% SCF, β-estradiol (0.5μM)).

##### In vitro *differentiation of ER-Hoxb8 progenitors into neutrophils*

ER-Hoxb8 progenitors were rinsed 2x in PBS to get rid of residual β-estradiol before plating in differentiation medium (RPMI-GlutaMax, 10% FBS, 1% Pen/Strep, 1% SCF). In the absence of estrogen, the cells undergo between 3-5 cell divisions and terminally differentiate into neutrophils.

##### In vitro *education of ER-Hoxb8 neutrophils into TANs*

TME supernatant (TME-sup) was produced *ex vivo* from orthotopic KP1.9 tumors following an established protocol.^83^ ER-Hoxb8 neutrophils were cultured at 5 x 10^5^ cells/ml in TME-sup for a maximum of three days to differentiate into TANs under normal or hypoxic conditions (1% oxygen level) and used for downstream analysis.

##### *In vivo* cell fate mapping of ER-Hoxb8 neutrophil progenitors

For *in vivo* transfer of ER-Hoxb8 neutrophil progenitors, C57BL/6J or Cas9/GFP mice were irradiated by a low-dose 4.5Gy using an X-Ray X-Strahl irradiator the night before cell transfer (day 0), because ER-Hoxb8 cells fail to engraft in the fully occupied hematopoietic niches of non-irradiated recipients (our own data,^84^). In the following two days (day 1 and 2), mice received repeated injections of 20 x 10^6^ CD45.1^+^ ER-Hoxb8 neutrophil progenitors i.v. (first dose in the morning, second dose in the evening). Transferred CD45.1^+^ neutrophils were analyzed 5 days later (day 7).

##### *In vitro* gene-editing of ER-Hoxb8 neutrophil progenitors via CRISPR/Cas9

To generate NTC, *Ccl3*-knockout (*Ccl3^ko^*), *Ccr1^ko^*, and *Ccl3-*overexpressing (*Ccl3^oe^*) ER-Hoxb8 neutrophils, we generated ER-Hoxb8 neutrophil progenitors from Cas9/GFP CD45.1^+^ STEM mice. These progenitors, which constitutively express Cas9/GFP, were transduced with *Ccl3* sgRNA, *Ccr1* sgRNA, NTC sgRNA, or *Ccl3^oe^* lentivectors (detailed in Lentiviral vector library) following the protocol described in “In vivo gene-editing of endogenous neutrophils”, while during spin-infection the cells were spiked with 4 μg/ml polybrene. These vectors co-expressed an mCherry reporter and puromycin resistance gene. Successfully transduced cells were selected in the presence of puromycin (10 μg/ml, Sigma Aldrich, #P8833) for a maximum of 7 days and were isolated based on mCherry expression by FACS. The efficiency of *Ccl3* deletion was confirmed by NGS.

#### Lentiviral vector library

For the design of NTC, *Ccl3*, and *Ccr1* sgRNAs, we used the CRISPick platform of the Broad Institute (https://portals.broadinstitute.org/gpp/public/analysis-tools/sgrna-design) and established protocols by the Zhang lab.^85,86^ The sequences of the sgRNAs were as follows: NTC sgRNA1, CATACCCGCGCCGTGACTCC; *Ccl3* sgRNA1, AACTTACATGACACCTGGCT; *Ccl3* sgRNA2, TAGTCAAC GATGAATTGGCG; *Ccr1* sgRNA1, AGATCTCACTGTATAAACCC; *Ccr1* sgRNA2, GTCATGATAATCTGCTATGC. These sgRNAs were cloned into the pKLV2-U6gRNA5(BbsI)-PGKpuro2AmCherry-W lentivector (Addgene, #67977) using an established protocol by the Zhang lab^85,86^ and a FastDigest Bpil restriction digest (Thermo Scientific, #FD1014). In addition, NTC sgRNA1 and *Ccl3* sgRNA1 were cloned into the pxpr_036_hCD19-reporter lentivector, which was provided by the Manguso lab (Broad Institute) and expresses a truncated form of human CD19, using established protocols by the Manguso lab and a BsmBI-v2 restriction digest (NEB, #R0739). We then generated lentiviruses by transfecting HEK293NT cells with lentivectors, packaging and envelope plasmids (Addgene #12263, #14888) using the FuGENE Transfection Reagent and protocol (Promega, #E2311). Lentiviruses with the pLV[Exp]-EGFP/Puro-EF1A>mCherry lentivector designed to overexpress *Ccl3 (Ccl3^oe^* lentivector: pLV[Exp]-CMV>mCcl3[NM_011337.2]:WPRE-mPGK>Puro:T2A:mCherry*)* were purchased from VectorBuilder Inc. Lentiviruses were then used to infect either ER-Hoxb8 neutrophil progenitors (mCherry^+^ lentivectors) or LSKs (hCD19^+^ lentivectors).

#### *In vivo* co-injection experiments of tumor cells with neutrophils

KP1.9 tumor cells were co-injected with either NTC or *Ccl3^ko^* or *Ccr1^ko^* ER-Hoxb8 TANs (Cas9/GFP^+^ CD45.1^+^) subcutaneously (s.c.) into the flank of Cas9/GFP^+^ mice. TANs were derived from a 3-day culture of WT or *Ccl3^ko^* ER-Hoxb8 neutrophils in TME-sup and FACS sorted based on cell surface marker expression (CD45.1^+^CD11b^+^ Ly-6G^+)^ as detailed in the “Flow Cytometry” methods section. Tumor cells (2×10^5^) and the respective neutrophil population (8×10^5^) were mixed in 50 μl 1 x PBS before s.c. injection (1:4 ratio). Tumor growth was recorded over time with a digital caliper and tumor volumes were defined as π/6 x length x width^2^.

#### Next-generation sequencing

Genomic DNA was extracted using the AllPrep DNA/RNA Mini Kit (Qiagen, #80204). *Ccl3* cut sites were amplified via PCR using the Phusion High-Fidelity PCR Master Mix (NEB, #M0531S) and the following primer sets at 10 μM: *Ccl3* sg1 Fwd Set1 5’-ACCCTCTCTTACACTGTTCCT-3’; *Ccl3* sg1 Rev Set1 5’-CTTCCTCGCCTCTTAAGTTGTT-3’; *Ccl3* sg2 Fwd Set1 5’-GCACG AGAACAGAACTTACATGAC-3’; *Ccl3* sg2 Rev Set1 5’-GCACCTTCCTCGCCTCTT-3’; *Ccl3* sg2 Fwd Set2 5’-AAAGCACG AGAACAGAACTT-3’;*Ccl3* sg2 Rev Set2 5’-TTAGCCTTCCAACTCCCA-3’ (IDT). PCR products were purified with the QIAquick PCR Purification Kit (Qiagen, #28106) and the resulting amplicons were characterized using a Qubit (Thermo Fisher) instrument. Amplicons were sequenced using Illumina next-generation sequencing at the Massachusetts General Hospital Center for Computational and Integrative Biology DNA Core (https://dnacore.mgh.harvard.edu). Paired end reads were merged using FLASH^87^ and CRISPR-induced mutations were analyzed using a custom pipeline based on the pattern-searching algorithm CRISPRpic.^88^

#### Cytospin of ER-Hoxb8 neutrophils

Cytospins were performed using a Shandon Cytospin 4 centrifuge (Thermo Fisher Scientific). 5 x 10^4^ ER-Hoxb8 neutrophils were centrifuged onto Shandon coated microscope Cytoslide (Thermo Fisher Scientific) and dried overnight at RT. Cells were then fixed in 4% formaldehyde-buffered solution (optional, cells can be stained after air-drying without fixation) and stained with Giemsa using the Thermo Scientific Shandon Varistain Gemini ES Automated Slide Stainer. Image documentation was performed using the NanoZoomer 2.0-RS slide scanner system (Hamamatsu) and the NanoZoomer Digital Pathology software.

#### Flow cytometry

Cell suspensions were stained for dead cells with Zombie Aqua or Zombie UV or Zombie NIR (BioLegend, #423101, 423107, 423105, respectively) for 20 minutes at RT. In some experiments, 7-AAD (BioLegend, #420403) or Propidium iodide (Invitrogen, #P1304MP) were used to exclude dead cells following the supplier’s protocol. Cells were incubated with Fc Block TruStain FcX (Clone 93, BioLegend) in PBS with 0.5% BSA for 10 minutes on ice, and stained with fluorochrome-labeled Abs for 20 minutes on ice (listed in key resources table). For the detection of *Ccl3* RNA levels, we used the PrimeFlow RNA assay kit and RNA probes from Invitrogen (#88–18005-210) following the supplier’s protocol. For identification of apoptotic cells, we stained for Annexin V following the supplier’s protocol (BioLegend, #640923). Cells were quantified using Precision Count Beads (BioLegend). Flow cytometry data were acquired on a Symphony A5 instrument (BD Bioscience) and analyzed with FlowJo software (v10.5.3; BD Biosciences). For sorting, cells were isolated on a FACS Aria II SORP (BD Biosciences) or a MoFlo Astrios EQ (Beckman Coulter) sorter.

#### Histology

##### Immunofluorescence (IF) of mouse tissue

KP1.9 tumor-bearing lungs were perfused post-mortem by PBS injection through the right ventricle of the heart. Then lungs were excised and placed in ice cold PBS. Of note, for hypoxyprobe (pimonidazole) stain, mice were injected i.v. (tail vein injection) with 60 mg/kg pimonidazole (hypoxyprobeTM-1, HP3–100Kit, Hpi) 90 minutes before sacrifice. Tissues were then fixed with 4% paraformaldehyde for 4 hours, dehydrated with 20% sucrose in PBS overnight, and finally embedded in OCT and cryopreserved. Tissue sections of 8 μm were prepared using a cryostat, permeabilized using 0.3% Triton X-100 in PBS for 5 minutes, and blocked using a buffer containing 1% bovine serum albumin (BSA) and 10% FBS for 30 minutes. Sections were stained with primary Abs diluted in blocking buffer overnight at 4°C in a dark humidified chamber and subsequently with fluorochrome conjugated secondary Abs diluted in blocking buffer for 30 minutes. Finally, DAPI staining was used to identify cell nuclei and sections were imaged using a Zeiss Axioscan 7 (Zeiss). The Abs used are listed in key resources table. For H&E stain, a standard protocol was used, and sections were imaged using a Zeiss Axioscan 7 (Zeiss). Images were analyzed using the QuPath v0.4.0 Digital Pathology software.

##### RNAscope of mouse tissue

RNAscope Multiplex Fluorescent V2 assay (Bio-techne, #323110) was performed according to supplier’s protocol on 10 μm sections from 4% paraformaldehyde-fixed, sucrose-cryoprotected frozen KP1.9-lung bearing tissues. Samples were incubated with Protease III for 15 minutes at 40°C. Probe-Mm-*Ccl3* (Bio-techne, #319471), Mm-*Ppib* (Bio-Techne, #313911) as positive control or Mm-*Dapb* (Bio-techne, #310043) as negative control were hybridized at 40°C for 2 hours. The signal was revealed with TSA Opal570 (1:1500, Akoya Biosciences, #FP1488001KT). After 30 minutes blocking with 1% BSA, tissues were incubated with primary goat anti-mouse MPO Ab overnight at 4°C. The next day, after incubation with secondary Ab (donkey anti-goat AF750), tissues were counterstained with DAPI and mounted with Prolong Diamond Antifade Mounting (Thermo Fisher, #P36965). Abs are listed in key resources table. Sections were imaged using a Zeiss Axioscan 7 (Zeiss). For analysis of *Ccl3^+^* TAN distribution in hypoxic areas, two consecutive tissue sections were prepared due to technical limitations of the RNAscope workflow: one stained for *Ccl3* and MPO using RNAscope, and the other for detecting pimonidazole localization by IF. KP1.9 tumor regions and parenchyma were annotated in lung tissue samples and cell segmentation within the annotated regions was performed using QuPath’s cell detection algorithms. Random forest classifiers were trained within QuPath to identify cells positive for pimonidazole, *Ccl3*, and MPO. Representative visualizations were generated using the QuPath v0.4.0 Digital Pathology software.

#### RNA isolation and qRT-PCR (qPCR)

Hox8-derived neutrophils and TANs were sorted either by MACS with anti-Ly6G magnetic beads following the provider’s protocol (Miltenyi Biotec, #130-120-337) or by FACS as CD45.1^+^CD11b^+^Ly-6G^+^ cells, and total RNA was isolated using miRNAeasy Mini kit (Qiagen, #217004) or RNeasy Plus Mini kit (Qiagen, #74134) following the supplier’s protocol. cDNA was synthesized employing the SuperScript IV VILO cDNA Synthesis kit (Invitrogen/Thermo Fisher Scientific, #11754250) according to the supplier’s protocol and quantitative real-time PCR (qRT-PCR, qPCR) was performed on a QuantStudio 6 Flex Real-Time PCR system (Thermo Fisher Scientific) by using the following TaqMan gene expression assays (Applied Biosystems/Thermo Fisher Scientific): Mm00441259 *Ccl3*, Mm00437783 *Bcl2l1,* Mm99999915 *Gapdh*. Expression levels of *Ccl3* and *Bcl2l1* were normalized to *Gapdh* (ΔCt).

#### Bulk RNA sequencing

Lung WT or *Ccl3^ko^* and blood WT ER-Hoxb8 neutrophils were isolated from orthotopic KP1.9 lung tumor-bearing mice by FACS as live 7-AAD^−^CD45.2^−^CD45.1^+^CD11b^+^Ly-6G^+^ cells and RNA was extracted in 75 μl RULT lysis buffer following the supplier’s protocol (RNeasy UCP Micro Kit, Qiagen, #73934). Library preparation and sequencing were performed by Novogene after using the SMARTer Low Input RNA Kit. Bulk mRNA sequences were aligned using STAR and RSEM. The log FPKM values were used in heatmap visualization and boxplot/two-sample t-test analyses.

#### *In vitro* pro-apoptotic treatment

5 x 10^5^ WT or *Ccl3^ko^* ER-Hoxb8 neutrophils were cultured in TME-sup from orthotopic KP1.9 lung tumors, increasingwith increasing concentrations (0nM, 0.1nM, 10nM and 100nM) of the H3-mimetic drugs A-1331852 or Navitoclax for three days. At day 3, the number of SiglecF^hi^ TANs was evaluated by flow cytometry and quantified relative to WT SiglecF^hi^ TANs cultured without drug (0nM).

#### Human studies

##### scRNAseq

Publicly available single-cell data were obtained from the respective repository. The unfiltered raw counts, when available, were used. If the raw sequence data were available, the unfiltered raw counts were produced using CellRanger software.

Analysis of single-cell RNA data were performed using R package scalpi (available from https://gitlab.com/pwirapati/scalpi). In scalpi, cell type classification was done automatically using the raw counts as the input, without the need of prior filtering and pre-processing, based on competing probabilities from the predicted multinomial logistic regression output. Low quality cell profiles were not filtered out based on criteria such as number of detected genes or total UMI, but by the cell type membership probability. The model includes a “debris” category which was obtained by artificially diluting well-annotated cell profiles (by resampling the read counts to have 20 times less coverage). This serves as competing category when classifying a new profile, thus performing the quality control and classification at the same time, by the multinomial logistic regression classifier. The model was trained on well-annotated data from Zilionis et al.^30^ that contains substantial number of tumor and blood neutrophils. After simultaneous cell filtering and classification based on the raw count profiles, the scRNA data were normalized and batch-corrected using mean scaling (also performed using the scalpi package). Dimensionality reduction was performed on log1p transformed data using singular-value decomposition. Hierarchical clustering and UMAP visualization were done on the reduced space using, respectively, nclust package (https://gitlab.com/pwirapati/nclust) and uwot R package (https://github.com/jlmelville/uwot).

Neutrophil subtypes (*SELL^high^*, *CCL3^neg/low^* and *CCL3^high^*; *Sell^high^*, *Ccl3^neg/low^* and *Ccl3^high^*) were identified manually from the branches of the hierarchical clustering tree of the combined single cell datasets (separately for human, Figure 2A and mouse, Figure 3A). Hierarchical clustering of combined human and mouse scRNA profiles were done at the level of the average profiles per subtype per species (Figure 4A).

The data and code are available from Zenodo with https://doi.org/10.5281/zenodo.17661115.

URL: (https://doi.org/10.5281/zenodo.17661115).

#### TotalSeq

11 patients from a previously published HNSCC dataset GSE234933^45^ were profiled with TotalSeq protocol, described in detail below, using antibodies (Abs) against a set of proteins including a neutrophil marker CD66b. Quantification of the Ab binding intensity was done using CellRanger 3.1.0, and the raw Ab capture counts were published as a supplemental file in updated Gene Expression Omnibus record GSE234933. Thresholding for present or absent values was based on the bimodality trough in the histogram of the logarithm of raw counts plus one.

##### Sample dissociation and dead cell removal

All samples in this study were rapidly dissociated within 30-45 minutes after the surgical procedure using the Human Tumor Dissociation kit from Miltenyi Biotec (cat# 130-095-929) with minor modifications to the protocol. First, tissue was placed into a 1.5 ml Eppendorf tube containing 420 μl DMEM, minced into small fragments (1-2 mm) using surgical scissors, followed by the addition of 42 μl of enzyme H, 21μl of enzyme R, 5μl of enzyme A (all provided in the kit), and 512 μl of DMEM (reaching a total volume of 1 ml). Second, the minced tissue was vortexed briefly for 2 seconds and was immediately placed in a thermomixer (Eppendorf; F1.5) for 15 minutes at 37°C, 350 rpm. Next, tissue was placed over a 50 μm filter (Sysmex; Cat# 04–004-2327), minced with a 1 ml syringe plunger, and was washed with 10 ml of DMEM containing 10% heat-inactivated FCS. After spinning the tube at 1500 rpm at 4°C for 5 minutes, the supernatant was removed, and ACK buffer (Gibco, cat# A1049201) was added to ensure the removal of red blood cells (RBCs). Following RBC lysis, the dissociated sample was resuspended in DMEM with 10% FCS and counted using a manual hemocytometer (Bright-line; Cat# 1492) to determine the number and viability of the cells. If the sample’s viability was below 70%, the EasySep Dead Cell Removal (Annexin V) kit (STEMCELL; cat# 17899) was used, with minor modifications to the protocol, allowing cell viability enrichment with a low input of cells. Briefly, cells were washed with 1 ml of room temperature (RT) isolation media (PBSx1 + 2% FCS + 1 mM CaCl2), resuspended in 100 μl, and moved into a PCR 8-strip well. Next, 10 μl dead cell removal (Annexin V) and 10 μl of biotin selection cocktails (provided with the kit) were added, gently mixed with the cell suspension, and incubated for 4 minutes at RT. After the first incubation, 20 μl of vortexed RapidSpheres and 60 μl of isolation media were added, mixed, and incubated for 3 minutes at RT. Lastly, the PCR 8-strip tube containing the cells was placed on a 10x Genomics magnetic separator (cat# 120250). After a 5-minute incubation at RT, the supernatant containing the viable cells was moved into a new 1.5 ml collection tube, span for 5 minutes at 1500 rpm, 4°C; resuspended in DMEM with 10% FCS (for 10x Genomics GEM generation and barcoding) or staining buffer (PBSx1 + FCS 2.5% + 2 mM EDTA; for TotalSeq-C staining), and counted using a manual hemocytometer.

##### TotalSeq-C staining

Dissociated cells were washed twice and resuspended with staining buffer (PBSx1 + FCS 2.5% + 2mM EDTA), followed by a 10-minute incubation at 4°C with TruStain FcX (Biolegend; Cat# 422301) at a 1:100 concentration. Next, cells were washed once with staining buffer and spun down at 1500 rpm, 4°C for 5 minutes. Equal concentrations (1:100) of each TotalSeq-C antibody were combined, mixed, and spun at 14,000 rpm for 5 minutes to remove any aggregates. Immediately after this step, cells were incubated for 20 minutes at 4°C with the TotalSeq-C antibody mix, washed twice with staining buffer, resuspended in DMEM with 10% FCS after the last wash, and filtered through a 50 μm filter (Sysmex; Cat# 04-004-2327) before counting and loading the cells on the 10x Chromium controller instrument.

##### Single-cell library generation and sequencing

Generation of single-cell whole transcriptome libraries was performed using the 10x Genomics Chromium Single Cell 5’ Library and Gel Bead Kit v1 chemistry (10x Genomics; Cat# 1000006) with Chromium Single Cell 5’ Feature Barcode Library kit (10x Genomics; Cat# 1000080) according to the manufacturer’s instructions. Quality assessment (QC) was performed for each sample after cDNA, gene, and cell surface protein library generation, using the Qubit dsDNA High Sensitivity kit (Invitrogen; Cat# Q32854) and the Agilent High Sensitivity BioA DNA kit (Agilent; Cat# 5067-4626), for concentration and size distribution evaluation, respectively. Only samples that have passed QC were sequenced on a NextSeq 500 sequencer (Illumina) using pair-end reads, with 26 reads for read 1 and 55 reads for read 2.

#### Pseudobulk analyses

Neutrophil deconvolution for bulk mRNA TCGA data was performed by deriving a glmnet predictor of neutrophil abundance based on pseudobulk profiles of scRNAseq datasets as features, and the known neutrophil frequency as the output. Survival analysis of the bulk mRNA data was done using Kaplan-Meier estimates and Cox regression analysis functions from the survival R package.

#### Pseudotime analyses

Pseudotime analysis was done by fitting a principal curve using the R package princurve to fit a smooth trajectory to the UMAP coordinates. The projection of the cell on the trajectory was directly obtained from the parameter lambda in the principal curve output. The mean pseudotime for a gene is obtained by averaging the lambda’s value of the cells, weighted by the log expression of the gene of the respective cells.

#### Gene set enrichment analyses

Gene set enrichment analyses (GSEA) were performed using fgsea and msigdb R packages. The gene-wise mean pseudotime were used as the continuous variable for ranking the genes in GSEA analysis. Only the top 20 leading genes common to human and mouse were shown in the heatmap visualization by average profile of neutrophil subtypes.

#### Histology

##### Immunohistochemistry (IHC) of human tissue

Four μm thick FFPE HNSCC sections of22 tumor samples (HN01, HN30, HN32, HN35, HN37, HN38, HN39, HN40, HN42, HN46, HN49, HN55, HN57, HN58, HN63, HN64, HN66, HN67, HN68, HN72, HN74, HN75) matching the tumors analyzed by scRNAseq (Bill et al.^45^ cohort) were stained for neutrophils by IHC. FFPE sections were dried at 55°C on a heating block overnight, then cooled down to RT for at least 1 hour, and treated with xylene/ethanol for deparaffinization. Hematoxylin & Eosin (H&E) stain was performed using a standard protocol. Neutrophils were stained by IHC using anti-CD66b Ab; slide pretreatment, reagents and the protocol used for staining were provided with the EnVision Flex Mini Kit, High pH (Agilent Dako, #K8023). The entire tissue cross-sections were imaged using a Zeiss Axioscan 7 (Zeiss). Images were analyzed in the QuPath v0.4.0 Digital Pathology software. The tumor area was identified on an H&E-stained adjacent tissue section by a board certified pathologist (P.M.S.).

##### RNAscope of human tissue

For combined RNA-ISH-IF (RNAscope) analysis, samples HN1, HN17, HN22, HN26, HN30, HN38, HN49, HN59, HN62, HN68, and HN74 with differences in TAN abundance (defined by scRNAseq analysis from Bill et al.^45^ cohort) were selected. Four μm thick sections from FFPE blocks were used and an *in situ* hybridization (ISH) with co-immunofluorescence staining (IF) was performed following the supplier’s protocol of the RNAscope Multiplex Fluorescent Reagent Kit v2 assay (Advanced Cell Diagnostics, #323100). Combined ISH and Ab staining with Opal dyes (Akoya Biosciences) was performed manually for ISH and by employing an automated Ventana Discovery Ultra Staining module (Ventana, Roche) for IF. Multiplex staining consisted of multiple rounds of staining. Each round included endogenous peroxidase quenching (Discovery Inhibitor, Roche, #760–4840), non-specific sites blocking for8 minutes (Discovery Goat IgG and Discovery Inhibitor, Roche, #760–6008), primary Ab incubation, secondary HRP-labeled Ab incubation for 16 minutes (OmniMap anti-rabbit HRP, Roche, #760–4311 or OmniMap anti-mouse HRP, Roche, #760–4310), OPAL™ reactive fluorophore detection (Akoya Biosciences, Marlborough, MS, USA), that covalently labels the primary epitope, and Ab heat denaturation (Discovery CC2, Roche, #950–223). The following combinations of RNAscope probe (purchased from Bio-techne) and Abs (listed in key resources table) were employed. All Opal dyes were purchased from Perkin Elmer AG. Probe-Hs-*CCL3* probe (#455331) with Opal690 (#FP1497001KT), anti-human MPO Ab with Opal520 (# FP1487001KT), anti-human GLUT-1 with Opal570 (FP1488001KT), and anti-human pan-cytokeratin Ab with Opal620 (# FP1495001KT).

Nuclei were visualized by final incubation with Spectral DAPI (1/10, #FP1490, Akoya Biosciences). The slides were mounted with fluorescence mounting medium (#S3023, Agilent Dako) and stored in the dark at 4°C until scanned within 48 hours. Images were acquired on the Vectra Polaris automated imaging system (Akoya Biosciences), and qualitatively analyzed using the inForm Analysis software (Akoya Biosciences) and the spati package (https://gitlab.com/pwirapati/spati) detailed in Bill et al.^45^ for spatial analysis. The tumor area was identified on an H&E-stained adjacent tissue section by a board-certified pathologist (P.M.S.).

### QUANTIFICATION AND STATISTICAL ANALYSIS

Statistical analyses were performed using either GraphPad Prism software (v10) or in R statistical software (https://www.R-project.org). Statistical significance was at the level of p < 0.05, with appropriate multiple testing applied depending on the context. Other details of statistical methods for specific analyses are described in figure legends.

## Supplementary Material

Supplemental S1-S9, Tables S1 and S2

Supplemental information can be found online at https://doi.org/10.1016/j.ccell.2026.01.006.

## Figures and Tables

**Table:** KEY RESOURCES TABLE

REAGENT or RESOURCE	SOURCE	IDENTIFIER
Antibodies		
Mouse monoclonal anti-mouse CD45.1 (clone A20), AF700	BioLegend	Cat#110723; Clone A20; RRID: AB_493732
Mouse monoclonal anti-mouse CD45.1 (clone A20), PE-Cy7	BD Biosciences	Cat#560578; Clone A20; RRID: AB_1727488
Mouse monoclonal anti-mouse CD45.2 (clone 104), AF700	Invitrogen	Cat#56045482; Clone 104; RRID: AB_657752
Mouse monoclonal anti-mouse CD45.2 (clone 104), BV421	BioLegend	Cat#109831; Clone 104; RRID: AB_10900256
Rat monoclonal anti-mouse CD45 (clone 30-F11), BUV805	BD Biosciences	Cat#748370; Clone 30-F11; RRID: AB_2872789
Rat monoclonal anti-mouse c-Kit (clone 2B8), PE-Cy7	Invitrogen	Cat#25117181; Clone 2B8; RRID: AB_469644
Rat monoclonal anti-mouse c-Kit (clone 2B8), APC	BioLegend	Cat#105812; Clone 2B8; RRID: AB_313221
Rat monoclonal anti-mouse CD11b (clone M1/70), APC-Cy7	BioLegend	Cat#101225; Clone M1/70; RRID: AB_830641
Rat monoclonal anti-mouse CD11b (clone M1/70), BV605	BioLegend	Cat#101257; Clone M1/70; RRID: AB_2565431
Rat monoclonal anti-mouse CD11b (clone M1/70), PE-Cy7	BioLegend	Cat#101216; Clone M1/70; RRID: AB_312799
Rat monoclonal anti-mouse CD11b (clone M1/70), PE	BioLegend	Cat#101208; Clone M1/70; RRID: AB_312791
Rat monoclonal anti-mouse Ly6C (clone HK1.4), BV711	BioLegend	Cat#128037; Clone HK1.4; RRID: AB_2562630
Rat monoclonal anti-mouse Ly6C (clone HK1.4), BV650	BioLegend	Cat#128049; Clone HK1.4; RRID: AB_2800630
Rat monoclonal anti-mouse Ly6C (clone HK1.4), BV421	BioLegend	Cat#128014; Clone HK1.4; RRID: AB_1732079
Rat monoclonal anti-mouse Ly6C (clone HK1.4), PE	BD Biosciences	Cat#553128; Clone HK1.4; RRID: AB_394644
Rat monoclonal anti-mouse Ly6G (clone 1A8), BV421	BioLegend	Cat#127612; Clone 1A8; RRID: AB_2251161
Rat monoclonal anti-mouse Ly6G (clone 1A8), APC	BioLegend	Cat#127614; Clone 1A8; RRID: AB_2227348
Rat monoclonal anti-mouse Ly6G (clone 1A8), BUV395	BD Biosciences	Cat#565964; Clone 1A8; RRID: AB_2739417
Rat monoclonal anti-mouse Ly6G (clone 1A8), PE-Cy7	BioLegend	Cat#127617; Clone 1A8; RRID: AB_1877262
Rat monoclonal anti-mouse Ly6G (clone 1A8), PE	BioLegend	Cat#127608; Clone 1A8; RRID: AB_1186099
Rat monoclonal anti-mouse F4/80 (clone BM8), PE	Invitrogen	Cat#12480180; Clone BM8; RRID: AB_465922
Rat monoclonal anti-mouse F4/80 (clone T45-2342), BUV737	BD Biosciences	Cat#749283; Clone T45-2342; RRID: AB_2873658
Rat monoclonal anti-mouse F4/80 (clone T45-2342), BV421	BD Biosciences	Cat#565411; Clone T45-2342; RRID: AB_2734779
Rat monoclonal anti-mouse Siglec-F (clone E50-2440), BV421	BD Biosciences	Cat#565934; Clone E50-2440; RRID: AB_2739398
Rat monoclonal anti-mouse Siglec-F (clone E50-2440), BUV395	BD Biosciences	Cat#740280; Clone E50-2440; RRID: AB_2740019
Rat monoclonal anti-mouse Siglec-F (clone E50-2440), PE	BD Biosciences	Cat#552126; Clone E50-2440; RRID: AB_394341
Rat monoclonal anti-mouse CCR1 (clone S15040E), PE	BioLegend	Cat#152507; Clone S15040E; RRID: AB_2800688
Rat monoclonal anti-mouse CCR1 (clone S15040E), APC	BioLegend	Cat#152503; Clone S15040E; RRID: AB_2629810
Armenian hamster monoclonal anti-mouse CCR5 (clone HM-CCR5), PE	BioLegend	Cat#107005; Clone HM-CCR5; RRID: AB_313300
Armenian hamster monoclonal anti-mouse CCR5 (clone HM-CCR5), APC	BioLegend	Cat#107011; Clone HM-CCR5; RRID: AB_2074528
Armenian hamster monoclonal anti-mouse CD11c (clone N418), BV711	BioLegend	Cat#117349; Clone N418; RRID: AB_2563905
Hamster monoclonal anti-mouse CD11c (clone HL3), PE	BD Biosciences	Cat#557401; Clone HL3; RRID: AB_396684
Rat monoclonal anti-mouse CD19 (clone 6D5), BV510	BioLegend	Cat#115546; Clone 6D5; RRID: AB_2562137
Rat monoclonal anti-mouse CD19 (clone 6D5), PE	BioLegend	Cat#152408; Clone 6D5; RRID: AB_2629817
Rat monoclonal anti-mouse CD90.2 (clone 53–2.1), BV711	BD Biosciences	Cat#740647; Clone 53–2.1; RRID: AB_2740336
Rat monoclonal anti-mouse CD90.2 (clone 53–2.1), PE	BD Biosciences	Cat#553006; Clone 53–2.1; RRID: AB_394545
Armenian hamster monoclonal anti-mouse CD3 (clone 145-2C11), BUV805	BD Biosciences	Cat#749276; Clone 145-2C11; RRID: AB_2873651
Rat monoclonal anti-mouse CD3 (clone 17A2), AF700	BioLegend	Cat#100215; Clone 17A2; RRID: AB_493696
Rat monoclonal anti-mouse CD8α (clone 53–6.7), BV605	BioLegend	Cat#100744; Clone 53–6.7; RRID: AB_2562609
Rat monoclonal anti-mouse CD4 (clone GK1.5), BUV496	BD Biosciences	Cat#612952; Clone GK1.5; RRID: AB_2813886
Mouse monoclonal anti-mouse NK1.1 (clone PK136), FITC	BioLegend	Cat#108705; Clone PK136; RRID: AB_313392
Mouse monoclonal anti-mouse NK1.1 (clone PK136), PE	BioLegend	Cat#108708; Clone PK136; RRID: AB_313395
Rat monoclonal anti-mouse B220/CD45R (clone RA3-6B2), PE	BD Biosciences	Cat#553090; Clone RA3-6B2; RRID: AB_394620
Rat monoclonal anti-mouse TER-119 (clone TER-119), PE	BioLegend	Cat#116208; Clone TER-119; RRID: AB_313709
Rat monoclonal anti-mouse CD49b (clone DX5), PE	BioLegend	Cat#108908; Clone DX5; RRID: AB_313415
Rat monoclonal anti-mouse CD127 (clone A7R34), PE	Invitrogen	Cat#12127182; Clone A7R34; RRID: AB_465844
Rat monoclonal anti-mouse Sca-1 (clone D7), BV421	BioLegend	Cat#108128; Clone D7; RRID: AB_2563064
Mouse monoclonal anti-human CD19 (clone HIB19), APC	BioLegend	Cat#302212; Clone HIB19; RRID: AB_314242
Rabbit polyclonal anti-pimonidazole	Hypoxyprobe (Hpi)	Cat#pab2627; RRID: AB_2934099
Goat polyclonal anti-mouse MPO	R&D Systems	Cat#AF3667; RRID: AB_2250866
Rabbit polyclonal anti-human MPO	Agilent Dako	Cat#A0398; RRID: AB_2335676
Mouse monoclonal anti-human Cytokeratin (clone AE1/AE3)	Agilent Dako	Cat#M3515; Clone AE1/AE3; RRID: AB_2132885
Rabbit polyclonal anti-human GLUT1	Sigma-Aldrich	Cat#HPA031345; RRID: AB_2673835
Rabbit monoclonal anti-cleaved Caspase-3 (clone 269518)	R&D Systems	Cat#MAB835; Clone 269518; RRID: AB_2243951
Goat anti-rabbit IgG, AF647	Invitrogen	Cat#A21246; RRID: AB_2535814
Goat anti-rabbit IgG, AF555	Invitrogen	Cat#A21428; RRID: AB_2535849
Donkey anti-goat IgG, DyLight 755	Invitrogen	Cat#SA5-10091; RRID: AB_2556671
Donkey anti-goat IgG, AF647	Invitrogen	Cat#A21447; RRID: AB_2535864
Anti-rabbit HRP	Roche	Cat#760–4311; RRID: AB_2811043
Anti-mouse HRP	Roche	Cat#760–4310; RRID: AB_2885182
Mouse monoclonal anti-human CD66b (clone G10F5)	Bio-Rad	Cat#MCA216T; Clone G10F5; RRID: AB_2291565
Rat monoclonal anti-mouse TruStain FcX (Clone 93)	BioLegend	Cat#101319; Clone 93; RRID: AB_1574973
Anti-human TruStain FcX	BioLegend	Cat#422301; RRID: AB_2818986
Bacterial and virus strains		
MSCVneo-HA-ER-Hoxb8	Wang et al.^43^	Addgene plasmid #222291; RRID: Addgene_222291
pKLV2-U6gRNA5(BbsI)-PGKpuro2AmCherry-W	Tzelepis et al.^77^	Addgene plasmid #67977; RRID: Addgene_67977
pxpr_036_hCD19	This paper	N/A
pCMV delta R8.2	This paper	Addgene plasmid #12263; RRID: Addgene_12263
VSV.G	Reya et al.^78^	Addgene plasmid #14888; RRID: Addgene_14888
pLV[Exp]-CMV>mCcl3[NM_011337.2]: WPRE-mPGK>Puro:T2A:mCherry	This paper	VectorBuilder plasmid #VB240515-1674bdc
Biological samples		
Human HNSCC FFPE samples	MGH, MEE	This study
Human HNSCC samples	MGH	GSE234933
Chemicals, peptides, and recombinant proteins		
A-1331852 inhibitor	MedChemExpress	Cat#HY-19741
Navitoclax inhibitor	MedChemExpress	Cat#HY-10087
Polybrene	Merck Millipore	Cat#TR-1003-G
Puromycin	Sigma-Aldrich	Cat#P8833
Fibronectin	Sigma-Aldrich	Cat#F-0895
FuGENE Transfection Reagent	Promega	Cat#E2311
β-estradiol	Sigma-Aldrich	Cat#50-28-2
Ficoll gradient	Cytiva	Cat#GE17-1440-02
RBC lysis buffer	Invitrogen	Cat#00-4333-57
ACK lysing buffer	Gibco	Cat#A1049201
Zombie Aqua	BioLegend	Cat#423101
Zombie UV	BioLegend	Cat#423107
Zombie NIR	BioLegend	Cat#423105
7-AAD	BioLegend	Cat#420403
Propidium iodide	Invitrogen	Cat#P1304MP
DAPI	Akoya Biosciences	Cat#FP1490
DAPI	ThermoFisher Scientific	Cat#D21490
Giemsa	Abcam	Cat#ab150670
Hematoxylin	Agilent Dako	Cat#CS70030-2
Fluorescence mounting medium	Agilent Dako	Cat#S3023
Prolong Diamond Antifade Mounting	ThermoFisher Scientific	Cat#P36965
Annexin V-BV421	BioLegend	Cat#640923
Pimonidazole (hypoxyprobeTM-1)	Hypoxyprobe (Hpi)	Cat#HP3-100
Recombinant mouse Flt3L	PeproTech	Cat#250-31L
Recombinant mouse IL-7	PeproTech	Cat#217-17
Recombinant mouse SCF	PeproTech	Cat#250-03
Recombinant mouse TPO	PeproTech	Cat#315-14
Recombinant mouse IL-6	STEMCELL	Cat#78052.1
Recombinant mouse IL-3	PeproTech	Cat#213-13
Critical commercial assays		
Human Tumor Dissociation kit	Miltenyi Biotec	Cat#130-095-929
EasySep Dead Cell Removal kit	STEMCELL	Cat#17899
10× Genomics Chromium Single Cell 5′ Library and Gel Bead Kit v1 chemistry	10× Genomics	Cat#1000006
Chromium Single Cell 5′ Feature Barcode Library kit	10× Genomics	Cat#1000080
Qubit dsDNA High Sensitivity kit	Invitrogen	Cat#Q32854
Agilent High Sensitivity BioA DNA kit	Agilent	Cat#5067-4626
AllPrep DNA/RNA Mini Kit	Qiagen	Cat#80204
QIAquick PCR Purification Kit	Qiagen	Cat#28106
EnVision Flex Mini Kit, High pH	Agilent Dako	Cat#K8023
PrimeFlow^™^ RNA Assay Kit	ThermoFisher Scientific	Cat#88-18005-204
RNAscope Multiplex Fluorescent Reagent Kit v2 assay	Advanced Cell Diagnostics	Cat# #323100
RNAscope Multiplex Fluorescent V2 assay	Bio-techne	Cat#323110
miRNAeasy Mini kit	Qiagen	Cat#217004
RNeasy Plus Mini kit	Qiagen	Cat#74134
RNeasy UCP Micro Kit	Qiagen	Cat#73934
SuperScript IV VILO cDNA Synthesis kit	Invitrogen	Cat#11754250
Deposited data		
Human and mouse neutrophil scRNA data compendium	This study	Zenodo: https://doi.org/10.5281/zenodo.17661115
Mouse *Ccl3^ko^* neutrophil bulk RNAseq	This study	Zenodo: https://doi.org/10.5281/zenodo.17661115
Human lung cancer (1) scRNA	Zilionis et al.,^30^ GEO	GEO: GSE127465
Human head and neck squamous carcinoma (1) scRNA and TotalSeq	Bill et al.,^45^ GEO	GEO: GSE234933
Human head and neck squamous carcinoma (2) scRNA	Kurten et al.,^46^ GEO	GEO: GSE164690
Human esophageal squamous cell carcinoma scRNA	Zhang et al.,^47^ GEO	GEO: GSE160269
Human lung cancer (2) scRNA	Salcher et al.,^32^ Zenodo	Zenodo: https://doi.org/10.5281/zenodo.6411867
Human pancreatic adenocarcinoma scRNA	Wang et al.,^35^ National Omics Data Encyclopedia	NODE: OEP003254
Mouse glioma scRNA	Alghamri et al.,^56^ GEO	GEO: GSE155275, GEO: GSE155276
Mouse liver cancer scRNA	Ramirez et al.,^57^ GEO	GEO: GSE216805
Mouse lung (1) cancer scRNA	Prieto et al.,^58^ GEO	GEO: GSE201247
Mouse lung (2) cancer scRNA	Gonzalez et al.,^59^ GEO	GEO: GSE217368
Mouse lung (3) cancer scRNA	Zilionis et al.,^30^ GEO	GEO: GSE127465
Mouse mammary (1) cancer scRNA	Ramos et al.,^60^ GEO	GEO: GSE192935
Mouse mammary (2) cancer scRNA	Li et al.,^62^ GEO	GEO: GSE189856
Mouse pancreas cancer scRNA	Caronni et al.,^61^ GEO	GEO: GSE216846
Experimental models: Cell lines		
Murine KP1.9 lung adenocarcinoma cell line derived from lung tumor nodules of male C57BL/6Kras^LSL–G12D/WT^;p53^Flox/Flox^ mouse	Alfred Zippelius	N/A
ER-Hoxb8 cell line derived from bone marrow cells of male C57BL/6J mouse	This paper	N/A
Cas9/GFP+ ER-Hoxb8 cell line derived from bone marrow cells of male Cas9/GFP CD45.1 STEM mouse	This paper	N/A
Experimental models: Organisms/strains		
Mouse: WT C57BL6/J	The Jackson Laboratory	JAX: 000664
Mouse: Gt(ROSA)26Sortm1.1(CAG-cas9*, -EGFP)Fezh/J (referred to as Cas9/GFP)	The Jackson Laboratory	JAX: 024858
Mouse: Gt(ROSA)26Sortm1.1(CAG-cas9*, -EGFP)Fezh/J CD45.1 STEM (referred to as Cas9/GFP CD45.1)	This paper	N/A
Mouse: Hemizygotes *Mrp8-Cre-eGFP* x hemi or homozygotes *Rosa26-LSL-Cas9-eGFP* (referred to as *Mrp8-Cas9/GFP*)	This paper	N/A
Mouse: *Ly6G-Cas9/GFP*	This paper	N/A
Oligonucleotides		
Primer *Ccl3* sg1 Fwd Set1 5′-ACCCTCTCTTACACTGTTCCT-3′	IDT	N/A
Primer *Ccl3* sg1 Rev Set1 5′-CTTCCTCGCCTCTTAAGTTGTT-3′	IDT	N/A
Primer *Ccl3* sg2 Fwd Set1 5′-GCACGAGAACAGAACTTACATGAC-3′	IDT	N/A
Primer *Ccl3* sg2 Rev Set1 5′-GCACCTTCCTCGCCTCTT-3′	IDT	N/A
Primer *Ccl3* sg2 Fwd Set2 5′-AAAGCACGAGAACAGAACTT-3′	IDT	N/A
Primer *Ccl3* sg2 Rev Set2 5′-TTAGCCTTCCAACTCCCA-3′	IDT	N/A
Oligo NTC sgRNA1, CATACCCGCGCCGTGACTCC	IDT	N/A
Oligo *Ccl3* sgRNA1, AACTTACATGACACCTGGCT	IDT	N/A
Oligo *Ccl3* sgRNA2, TAGTCAACGATGAATTGGCG	IDT	N/A
Oligo *Ccr1* sgRNA1, AGATCTCACTGTATAAACCC	IDT	N/A
Oligo *Ccr1* sgRNA2, GTCATGATAATCTGCTATGC	IDT	N/A
Recombinant DNA		
Lentivector pKLV2-U6sgRNA(NTC, *Ccr1* or *Ccl3*)PGKpuro2AmCherry-W	This paper	N/A
Lentivector pxpr_036_sgRNA (NTC or *Ccl3*)_hCD19	This paper	N/A
Lentivector pLV[Exp]-CMV>mCcl3[NM_011337.2]:WPRE-mPGK>Puro:T2A:mCherry	This paper	VectorBuilder plasmid #VB240515-1674bdc
Software and algorithms		
Custom analysis scripts	This paper	https://doi.org/10.5281/zenodo.17661115
Code used for scRNAseq analyses (R package *scalpi*)	This paper	https://gitlab.com/pwirapati/scalpi
Code used for spatial analyses (R package *spati*)	This paper	https://gitlab.com/pwirapati/spati
Gene Set Enrichment Analysis	R/Bioconductor	fgsea, msigdbr
CellRanger v4.x.x	10× Genomics	https://www.10xgenomics.com/support/software/cell-ranger/downloads
FlowJo v10	FlowJo, LLC	RRID_008520
GraphPad Prism v10	GraphPad Prism	RRID_002798
QuPath v0.5.0 Digital Pathology	Bankhead et al.^79^	RRID_018257
inForm 2.5.1 Automated Image Analyses	Akoya Bioscience	https://www.akoyabio.com/phenoimager/inform-tissue-finder/

## Data Availability

Custom analysis scripts and codes generated in this study are publicly available. Repositories are listed in the key resources table. Histology and flow cytometry data, single-cell AnnData objects, and code to reproduce the analyses and figures will be shared by the lead contact upon request. Any additional information required to reanalyze the data reported in this article is available from the lead contact upon request.

## Highlights

- Classifier recovers neutrophils across cancers for unified cross-dataset analysis
- CCL3 marks conserved terminal neutrophils with pro-tumor programs
- Neutrophil-derived CCL3 drives tumor growth via CCR1-dependent survival
- Hypoxia induces CCL3 and promotes the accumulation of CCL3^hi^ neutrophils
