# Supplementary material for: CCL3 is produced by aged neutrophils across cancers and promotes tumor growth
Source: Cancer Cell. Author manuscript; Available in PMC 2026 May 16. (PMC13179570; doi:10.1016/j.ccell.2026.01.006)
Supplement: Supplemental S1-S9, Tables S1 and S2 [file NIHMS2169884-supplement-Supplemental_S1-S9__Tables_S1_and_S2.pdf]

Cancer Cell, Volume *44*

Supplemental information

CCL3 is produced by aged neutrophils across cancers and promotes tumor growth

Evangelia Bolli, Pratyaksha Wirapati, Mehdi Hicham, Yuxuan Xie, Marie Siwicki, Florent Duval, Anne-Gaëlle Goubet, Máté Kiss, Béatrice Zitti, Thomas Zwahlen, Sheri Mcdowell, Ruben Bill, Simona Angerani, Camilla Engblom, Seth Anderson, Aiping Jiang, Oliver Hartley, David B. Sykes, Maja Jankovic, Nadine Fournier, Matthias Gunzer, David Tarussio, Stéphanie Tissot, Peter M. Sadow, William C. Faquin, Moshe Sade-Feldman, Ralph Weissleder, Sara Pai, François Mercier, Robert Manguso, and Mikaël J. Pittet

Supplementary Materials

Table of contents

- Figures S1 to S9
- Tables S1 to S2

Supplementary Figures

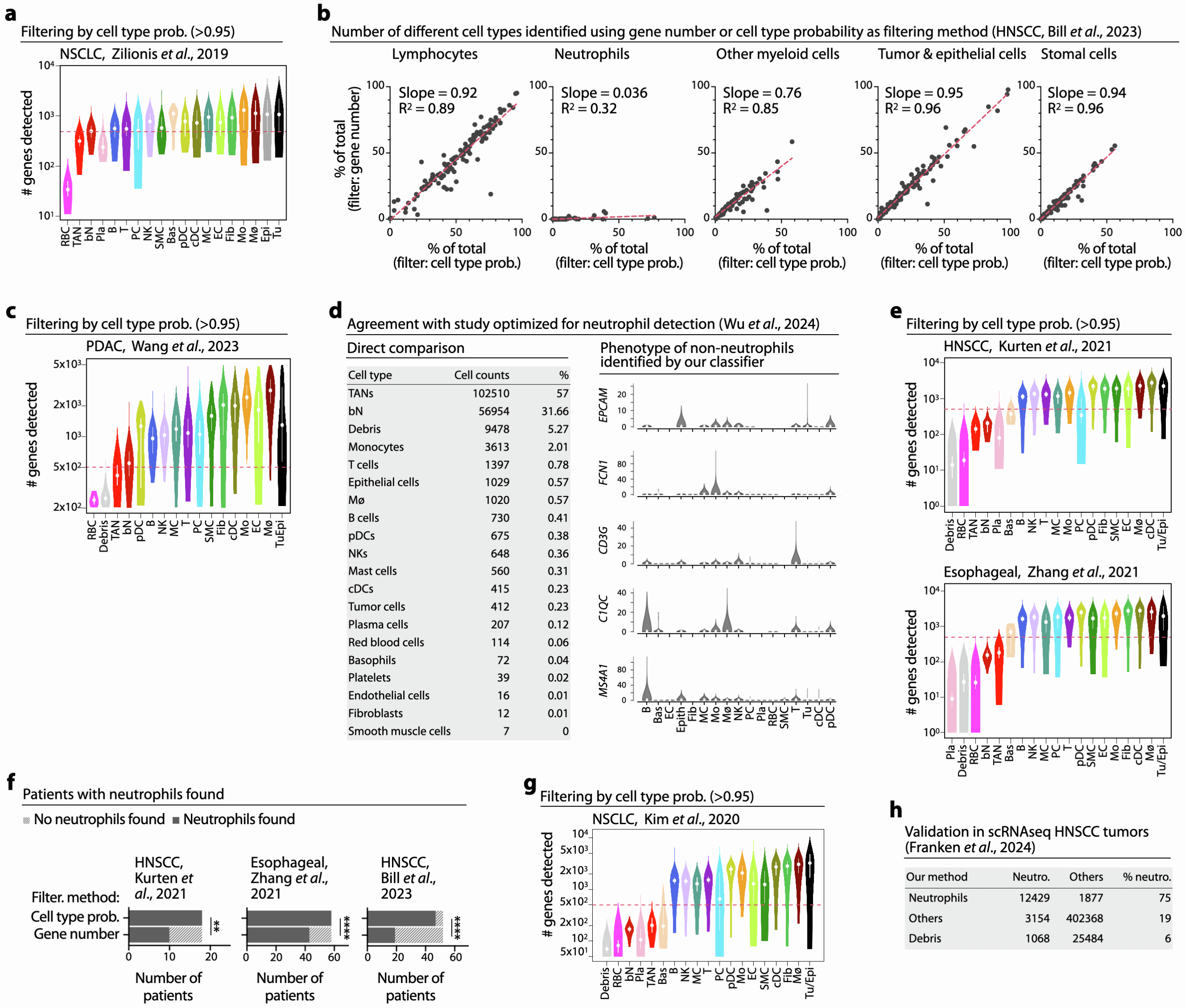

Cell type	Cell counts	%
TANs	102510	57
bN	56954	31.66
Debris	9478	5.27
Monocytes	3613	2.01
T cells	1397	0.78
Epithelial cells	1029	0.57
Mø	1020	0.57
B cells	730	0.41
pDCs	675	0.38
NKs	648	0.36
Mast cells	560	0.31
cDCs	415	0.23
Tumor cells	412	0.23
Plasma cells	207	0.12
Red blood cells	114	0.06
Basophils	72	0.04
Platelets	39	0.02
Endothelial cells	16	0.01
Fibroblasts	12	0.01
Smooth muscle cells	7	0

Our method	Neutro.	Others	% neutro.
Neutrophils	12429	1877	75
Others	3154	402368	19
Debris	1068	25484	6

Figure S1: Cell type probability-based scRNAseq filtering identifies neutrophils as independent predictors of cancer outcome, related to Figure 1.

(a) Cell filtering of Zilionis *et al*. scRNAseq dataset[S1] by cell type probability with the distribution of number of expressed genes shown. Dashed red lines represent a cutoff of >500 genes, typically used in filtering by gene number.

(b) Linear regression of number of different cell types (as % of total) filtered by cell type probability or gene number using our scRNAseq dataset[S2].

(c) Cell filtering of Wang *et al*. scRNAseq dataset[S3] by cell type probability with the distribution of number of expressed genes shown. Dashed red lines represent a cutoff of >500 genes, typically used in filtering by gene number.

(d) Left: The dataset from Wu *et al*.[S4] was filtered and include only RNA profiles from cells identified as neutrophils by the authors. We applied our classifier without modification or preprocessing to the raw count matrix of these data. The number of cells identified as a particular cell type is shown, as well the percentage relative to the total number of cells in the dataset. 89% of the cells were identified as either tumor or blood neutrophils (TAN or bN), and 5% as "Debris" (low quality data). Right: Marker gene expression in discrepant cell annotations. Cells labeled as neutrophils in Wu *et al*.[S4] but classified as non-neutrophil by our method display high expression of lineage-specific markers, including EPCAM (epithelial cells), FCN1 (monocytes), CD3G (T cells), C1QC (macrophages), and MS4A1 (B cells). These profiles indicate that the discrepant calls correspond to low-quality profiles or likely doublets.

(e) Cell filtering of two independent scRNAseq datasets[S5],[S6] by cell type probability with the distribution of number of expressed genes shown. Dashed red lines represent a cutoff of >500 genes, typically used in filtering by gene number.

(f) Comparison of number of patients with neutrophils found by applying either cell type probability or gene number approaches. Data are shown for three independent scRNAseq datasets[S2],[S5],[S6]. Chi-square, Fisher's exact test; **p<0.01, ****p<0.0001.

(g) Cell filtering of scRNAseq dataset from Kim *et al*. dataset[S7]. Cell filtering was done as in Figures S1a, c, and e, i.e. by cell type probability with the distribution of number of expressed genes shown. Dashed red lines represent a cutoff of >500 genes, typically used in filtering by gene number.

(h) Application of our classifier to Franken *et al*. dataset[S8]. We used the published raw data that have been filtered and annotated by the authors. The table shows the confusion matrix between our annotation (rows) and the original (columns). For simplicity all other cell types were pooled as "Others" and our "TAN" and "bN" are pooled as “Neutrophils".

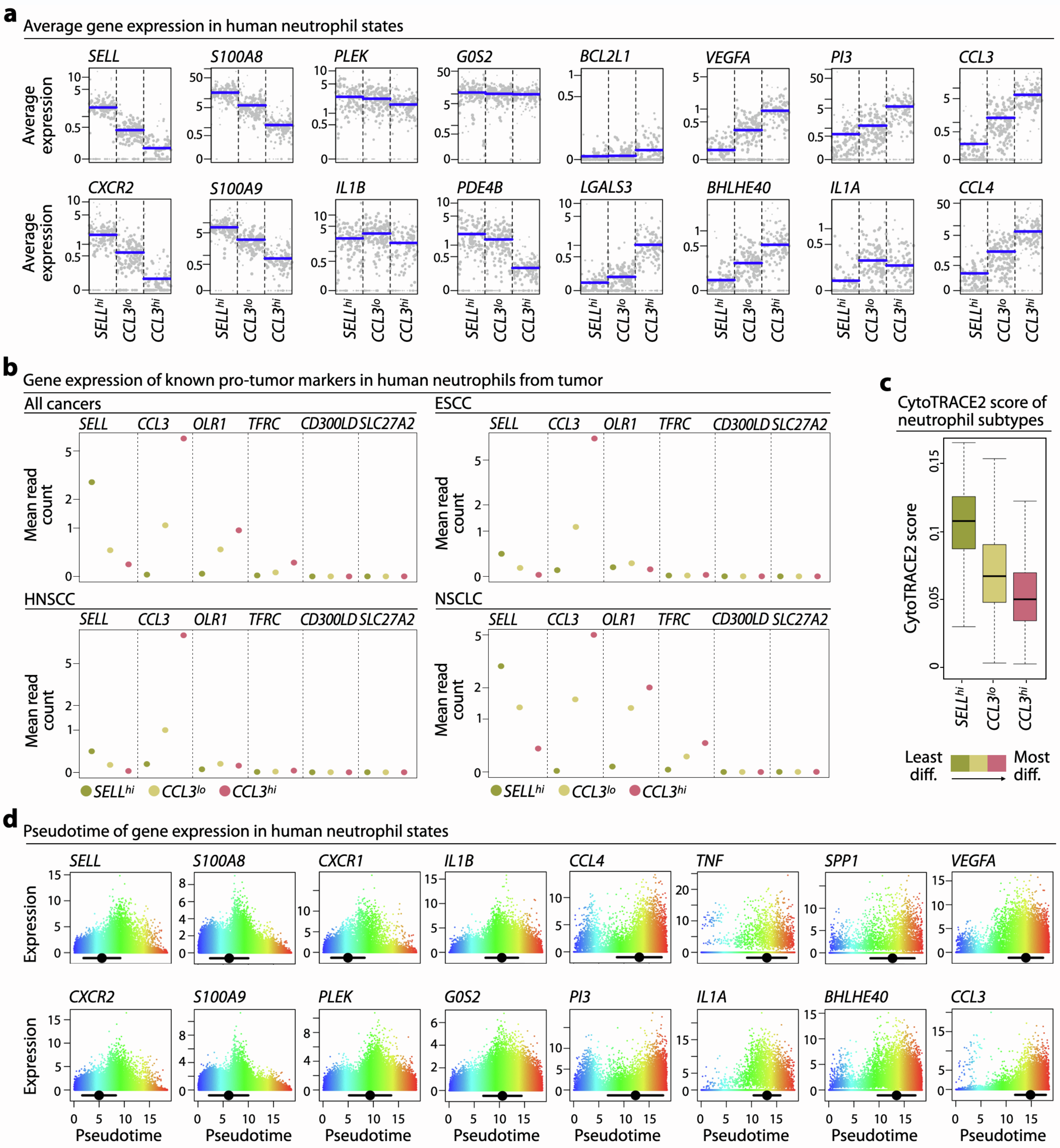

Figure S2: ***CCL3**^hi* **neutrophils can be found in diverse human tumors and represent an aged, pro-tumor state, related to Figure 2.**

(a) Average expression of representative genes that are differentially expressed in the three human neutrophil states. Data are shown for n = 157 patients, as introduced in Figure 2a.

(b) Mean read count of *SELL*, *CCL3* and known pro-tumor genes in the three tumor-derived human neutrophil states shown in all cancers or by cancer type. Data are shown for n= 152 total patients; n= 58 ESCC; n= 70 HNSCC; n= 24 NSCLC, as introduced in Figure 2a.

(c) Application of CytoTRACE2 algorithm to our neutrophil subtypes. The CytoTRACE2 method[S9], for calculating cell-development potential scores, was applied to our scRNAseq data. The distribution of the scores, stratified by our neutrophil subtypes, is shown as boxplots, revealing developmental potency from $SELL^{hi}$ to $CCL3^{lo}$ to $CCL3^{hi}$ neutrophil subtypes. Pairwise comparisons of the potency scores between groups (two-sided Wilcoxon test , p= 2.2e-16).

(d) Pseudotime of gene expression in human neutrophil states. For each gene, the expression-weighted mean pseudotime and its standard deviation were summarized (middle-black dots).

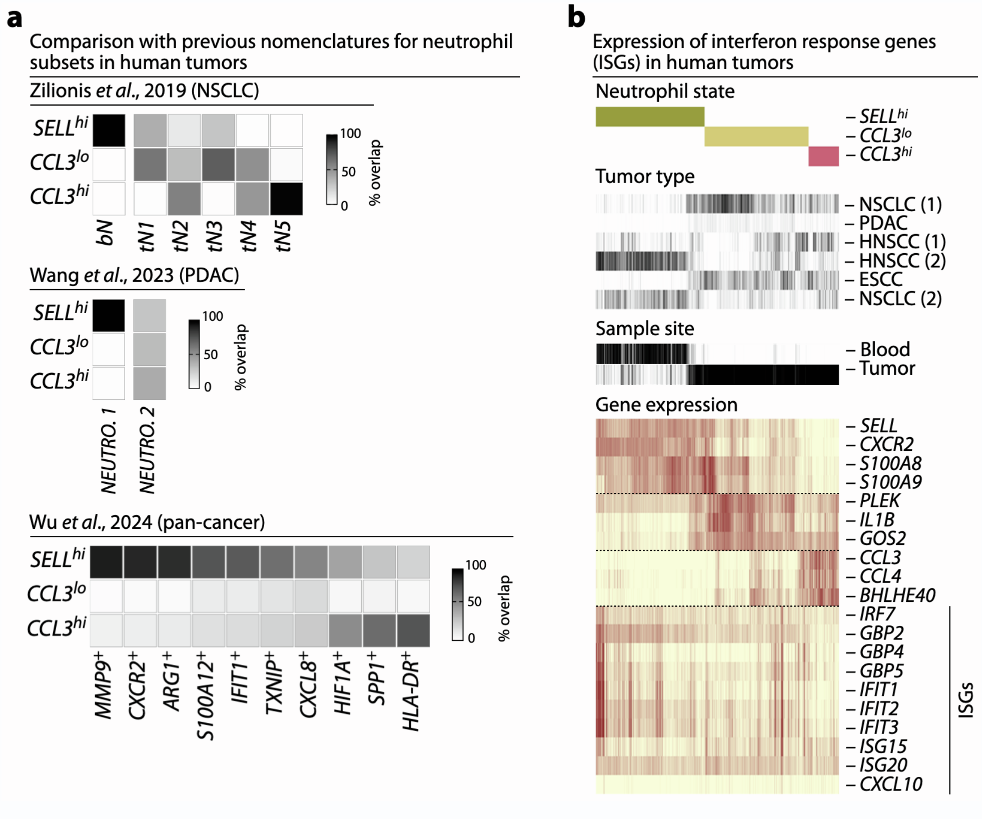

Figure S3: Comparison with previous nomenclatures for neutrophil states in human tumors, related to Figure 2.

(a) Comparison of our three-state TAN classification with previously published human TAN states from three independent studies[S1],[S3],[S4]. For each of our defined states, the percentage overlap with each published state was calculated.

(b) Activation of interferon response genes in human scRNAseq data. The heatmap, derived from Figure 2A, shows a subset of genes while maintaining the same cell order. Interferon response genes (ISGs) are shown at the bottom, from *IRF7* to *CXCL10*.

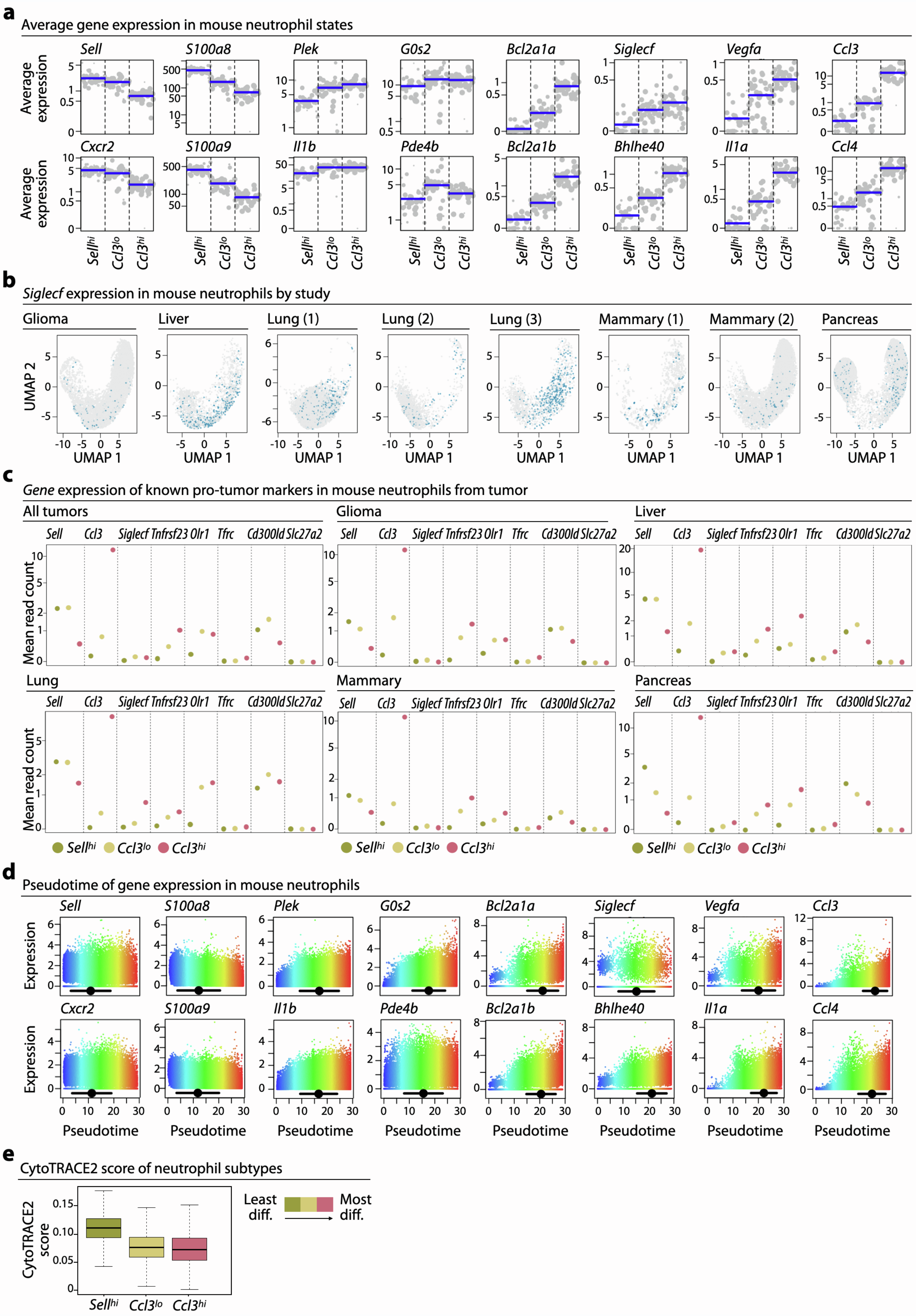
a
Average gene expression in mouse neutrophil states
Average expression
Sell
S100a8
Plek
G0s2
Bcl2a1a
Siglecf
Vegfa
Ccl3
Cxcr2
S100a9
Il1b
Pde4b
Bcl2a1b
Bhlhe40
Il1a
Ccl4
Sellhi
Ccl3lo
Ccl3hi
b
Siglecf expression in mouse neutrophils by study
Glioma
Liver
Lung (1)
Lung (2)
Lung (3)
Mammary (1)
Mammary (2)
Pancreas
UMAP 2
UMAP 1
c
Gene expression of known pro-tumor markers in mouse neutrophils from tumor
All tumors
Glioma
Liver
Lung
Mammary
Pancreas
Sell Ccl3 Siglecf Tnfrsf23 Olr1 Tfrc Cd300ld Slc27a2
Mean read count
Sellhi Ccl3lo Ccl3hi
d
Pseudotime of gene expression in mouse neutrophils
Expression
Pseudotime
e
CytoTRACE2 score of neutrophil subtypes
CytoTRACE2 score
Least diff.
Most diff.
Sellhi
Ccl3lo
Ccl3hi

Figure S4: ***Ccl3**^{hi}* **neutrophils can be found in diverse mouse tumors and represent an aged, pro-tumor state, related to Figure 3.**

(a) Average expression of representative genes that are differentially expressed in the three mouse neutrophil states. Data are shown for n = 39 mice, as introduced in Figure 3a.

(b) UMAPs of *Siglecf* expression in the three neutrophil states by study.

(c) Mean read count of *Sell*, *Ccl3* and known pro-tumor genes in the three tumor-derived mouse neutrophil states shown in all tumors or by tumor type. Data are shown for n = 39 total mice; n = 2 for glioma; n = 1 for liver; n = 10 for lung; n = 15 for mammary; n = 11 for pancreas, as introduced in Figure 3a.

(d) Pseudotime of gene expression in mouse neutrophil states. For each gene, the expression-weighted mean pseudotime and its standard deviation were summarized (middle-black dots).

(e) Application of CytoTRACE2 algorithm to our neutrophil subtypes. The CytoTRACE2 method[S9] for calculating cell-development potential scores was applied to our scRNAseq data. The distribution of these scores, stratified by our neutrophil subtypes, is shown as box plots, revealing developmental potency from *Sell*^{hi} to *Ccl3*^{lo} to *Ccl3*^{hi} neutrophils. Pairwise comparisons of the potency scores between groups (two-sided Wilcoxon test, p= 2.2e-16).

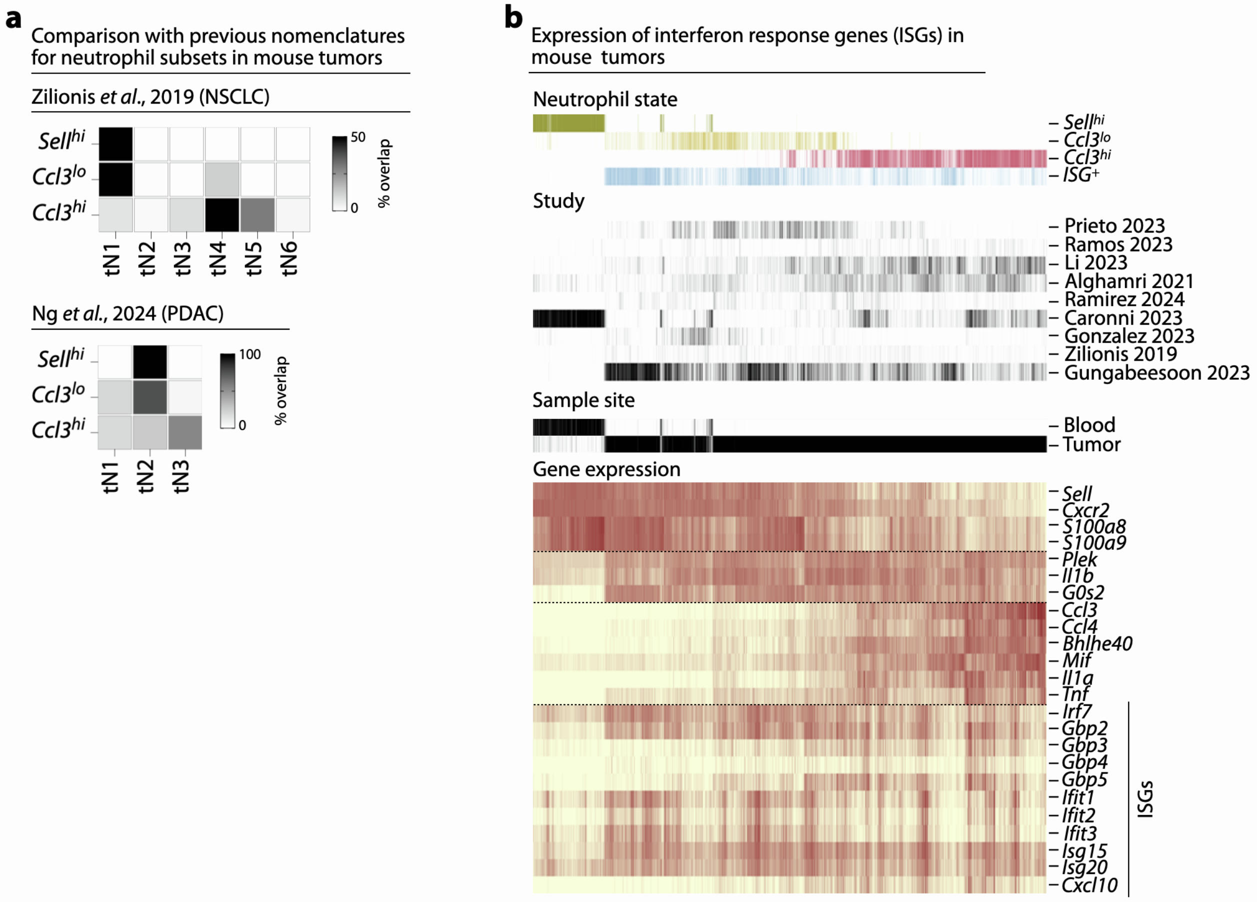

Figure S5: Comparison with previous nomenclatures for neutrophil states in mouse tumors, related to Figure 3.

(a) Comparison of our three-state TAN classification with previously published mouse TAN states from two independent studies[S1],[S10]. For each of our defined states, the percentage overlap with each published state was calculated.

(b) Activation of interferon response genes in mouse scRNAseq data. The heatmap is derived from Figure 3a, including the Gungabeesoon *et al.* dataset[S11], showing only selected genes, with the same ordering of the cells. Interferon response genes (ISGs) are shown at the bottom part, from *Irf7* to *Cxcl10*.

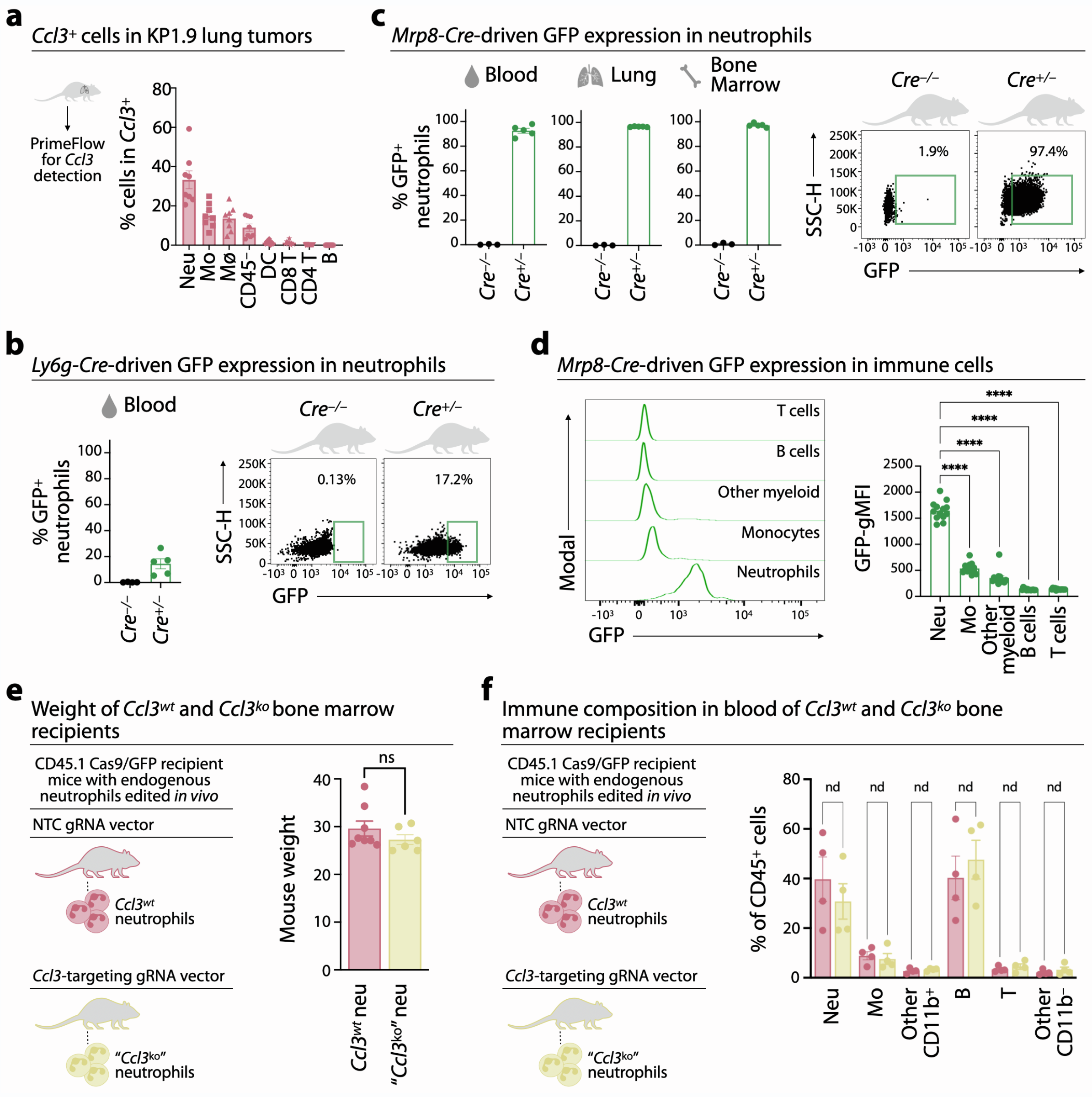

Figure S6: Neutrophil-derived CCL3 promotes tumor growth in mice, related to Figure 5.

(a) Proportion of various cell populations among *Ccl3*+ cells in KP1.9 orthotopic lung tumors, as measured by flow cytometry (PrimeFlow). n = 8 mice.

(b) Proportion of GFP+ in neutrophils from *Ly6g-Cas9/GFP* mice (*Ly6g-Cre+/– Cas9GFP+/–* or *Ly6g-Cre–/– Cas9GFP+/–*), as measured by flow cytometry. n = 3-5 mice per group.

(c) Proportion of GFP+ in neutrophils from *Mrp8-Cas9/GFP* mice (*Mrp8-Cre+/– Cas9GFP+/–* or *Mrp8-Cre–/– Cas9GFP+/–*), as measured by flow cytometry. n = 3-5 mice per group.

(d) GFP expression driven by *Mrp8-Cre*, measured as gMFI in various immune cell populations from *Mrp8-Cas9/GFP* mice (*Mrp8-Cre+/– Cas9GFP+/–*). n = 13 mice.

(e) Body weight of *Ccl3*^{wt} or "*Ccl3*^{ko}" bone marrow-reconstituted mice was measured 4 weeks post bone marrow transfer. n = 6-8 mice per group. Unpaired t-test.

(f) Immune cell composition in the blood of *Ccl3*^{wt} and "*Ccl3*^{ko}" bone marrow-reconstituted mice was evaluated 5 weeks post bone marrow transfer by flow cytometry. n = 4 mice per group. Multiple unpaired t-tests.

Bar graphs show mean ± SEM. ns, non significant, nd, no discovery=non significant.

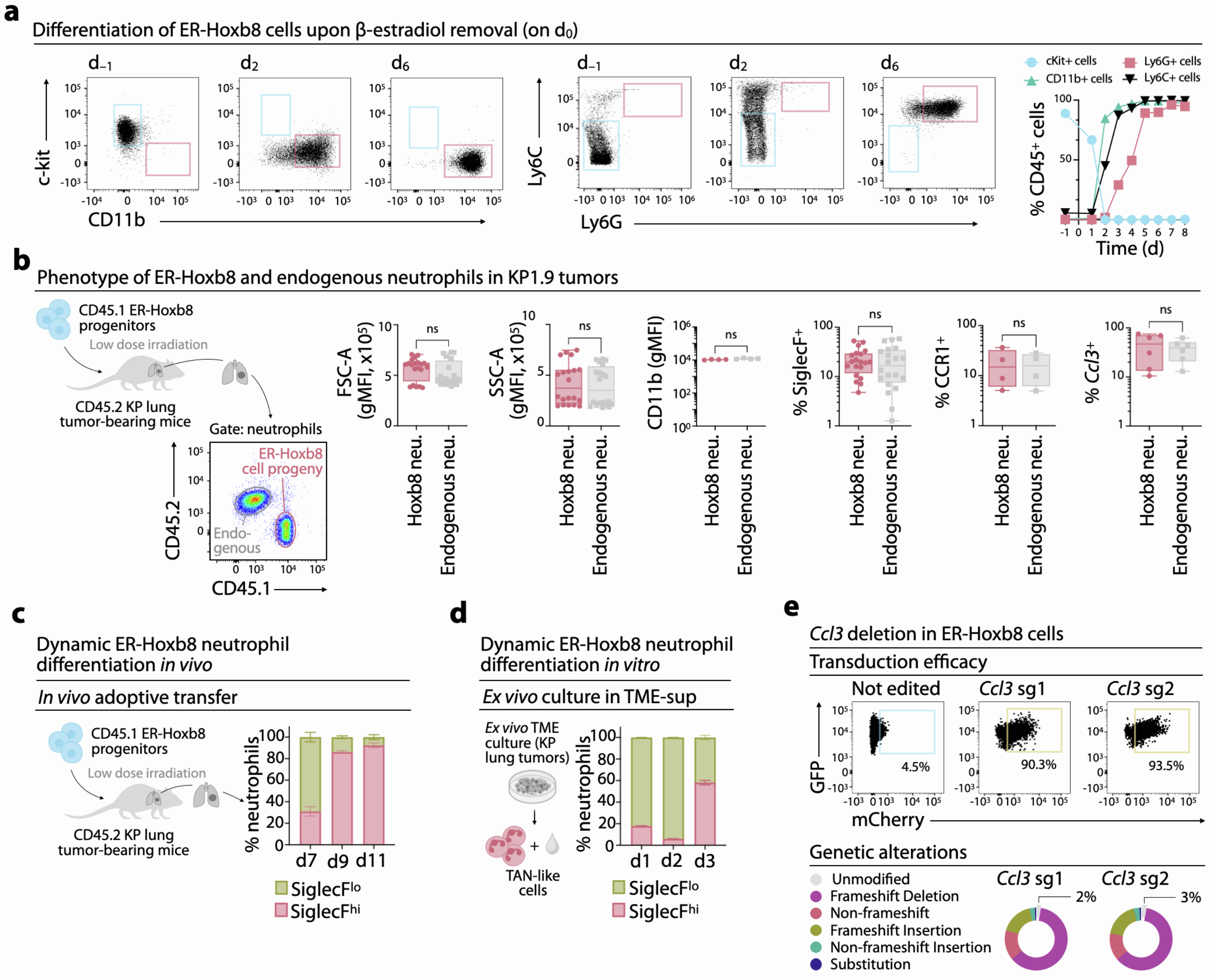

Figure S7: Neutrophil-derived CCL3 promotes tumor growth in mice, related to Figure 5.

(a) *In vitro* differentiation of ER-Hoxb8 neutrophil progenitors into fully mature CD45.1⁺ c-kit⁻ CD11b⁺ Ly6C^int Ly6G⁺ neutrophils over a 10-day differentiation period, assessed by flow cytometry.

(b) Schematic of CD45.1⁺ ER-Hoxb8 neutrophil progenitor transfer into C57BL6/J mice bearing orthotopic KP1.9 tumors (left). Phenotypic comparison of CD45.1⁺ ER-Hoxb8 TANs with endogenous CD45.2⁺ TANs from the same KP1.9-bearing lungs, evaluated by flow cytometry (right). FSC, SSC, and SiglecF analyses: n = 20 mice per group. Wilcoxon paired test. CD11b, CCR1, and *Ccl3* (PrimeFlow) analyses: n = 4-6 mice per group. Paired t-test.

(c), (d) Proportion of SiglecF^lo and SiglecF^hi cells in ER-Hoxb8 TANs measured by flow cytometry either at days 7-9-11 of *in vivo* transfer (c, n = 3-4 mice per group) or at days 1-2-3 of *in vitro* culture in TME-sup (d, n= 3 replicates per group).

(e) Transduction efficacy of *Ccl3* sg1- and *Ccl3* sg2-transduced CD45.1⁺ ER-Hoxb8 Cas9/GFP⁺ neutrophil progenitors, measured as the percentage of mCherry⁺ cells by flow cytometry (upper

panels). Transduction efficiency exceeded 90%. Gene editing efficacy of *Ccl3* in these cells was evaluated by next-generation sequencing, confirming effective *Ccl3* deletion (lower panels).

Box and whiskers plot (min to max) are presented in (b). Bar graphs in (c), (d) show mean ± SEM. ns, non significant.

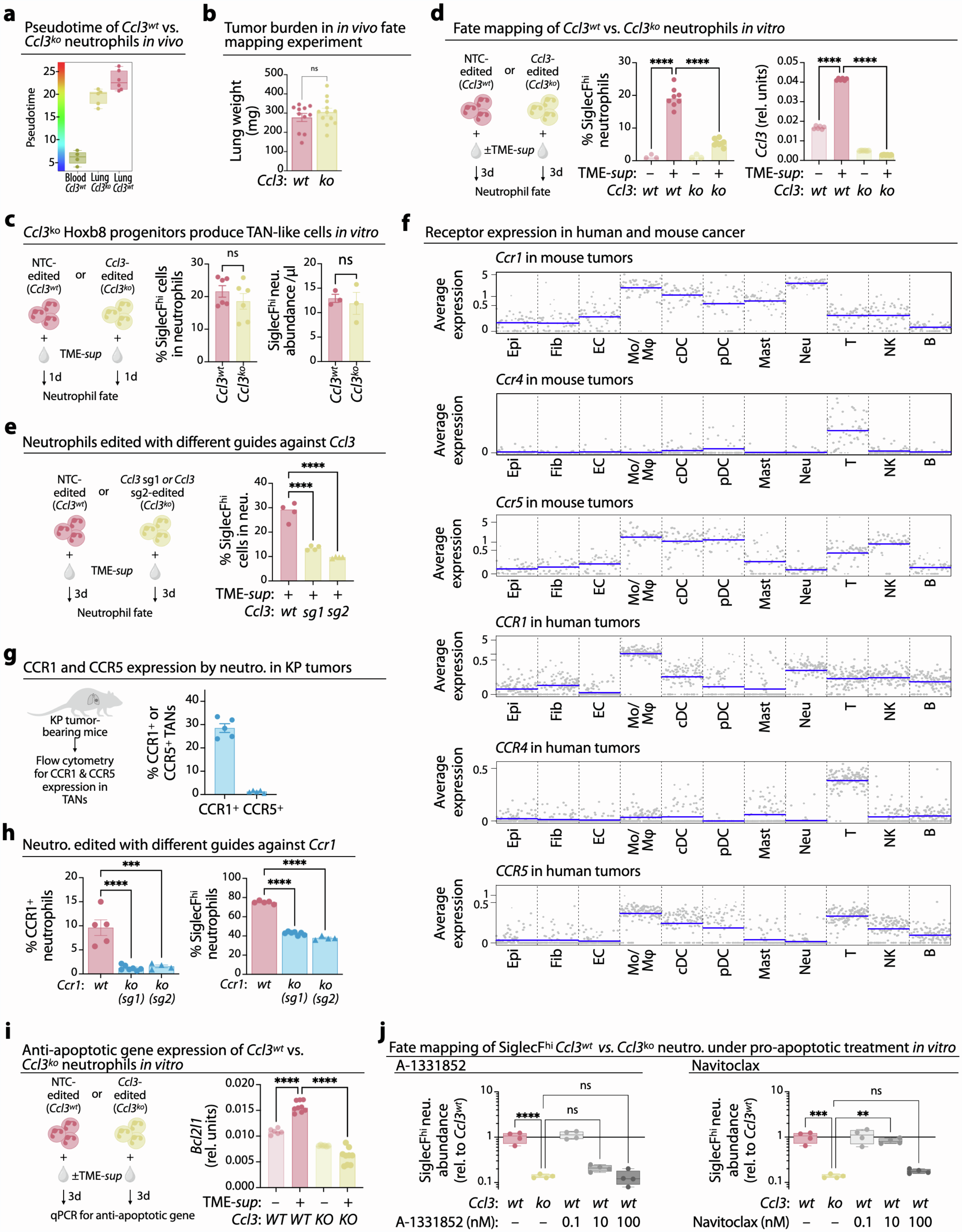
a
Pseudotime of Ccl3wt vs. Ccl3ko neutrophils in vivo
Pseudotime
Blood Ccl3wt
Lung Ccl3ko
Lung Ccl3wt
b
Tumor burden in in vivo fate mapping experiment
Lung weight (mg)
ns
Ccl3: wt ko
c
Ccl3ko Hoxb8 progenitors produce TAN-like cells in vitro
NTC-edited (Ccl3wt)
or
Ccl3-edited (Ccl3ko)
TME-sup
1d
Neutrophil fate
% SiglecFhi cells in neutrophils
SiglecFhi neu. abundance /µl
ns
Ccl3wt
Ccl3ko
d
Fate mapping of Ccl3wt vs. Ccl3ko neutrophils in vitro
NTC-edited (Ccl3wt)
or
Ccl3-edited (Ccl3ko)
±TME-sup
3d
Neutrophil fate
% SiglecFhi neutrophils
Ccl3 (rel. units)
****
TME-sup: – + – +
Ccl3: wt wt ko ko
e
Neutrophils edited with different guides against Ccl3
NTC-edited (Ccl3wt)
or
Ccl3 sg1 or Ccl3 sg2-edited (Ccl3ko)
TME-sup
3d
Neutrophil fate
% SiglecFhi cells in neu.
****
TME-sup: + + +
Ccl3: wt sg1 sg2
f
Receptor expression in human and mouse cancer
Ccr1 in mouse tumors
Ccr4 in mouse tumors
Ccr5 in mouse tumors
CCR1 in human tumors
CCR4 in human tumors
CCR5 in human tumors
Average expression
Epi
Fib
EC
Mo/Mφ
cDC
pDC
Mast
Neu
T
NK
B
g
CCR1 and CCR5 expression by neutro. in KP tumors
KP tumor-bearing mice
Flow cytometry for CCR1 & CCR5 expression in TANs
% CCR1+ or CCR5+ TANs
CCR1+ CCR5+
h
Neutro. edited with different guides against Ccr1
% CCR1+ neutrophils
% SiglecFhi neutrophils
****
***
Ccr1: wt ko (sg1) ko (sg2)
i
Anti-apoptotic gene expression of Ccl3wt vs. Ccl3ko neutrophils in vitro
NTC-edited (Ccl3wt)
or
Ccl3-edited (Ccl3ko)
±TME-sup
3d
qPCR for anti-apoptotic gene
Bcl2l1 (rel. units)
****
TME-sup: – + – +
Ccl3: WT WT KO KO
j
Fate mapping of SiglecFhi Ccl3wt vs. Ccl3ko neutro. under pro-apoptotic treatment in vitro
A-1331852
Navitoclax
SiglecFhi neu. abundance (rel. to Ccl3wt)
****
***
**
ns
Ccl3: wt ko wt wt wt
A-1331852 (nM): – – 0.1 10 100
Navitoclax (nM): – – 0.1 10 100

Figure S8: Neutrophil-derived CCL3 sustains pro-tumor neutrophil survival, related to Figure 6.

(a) Pseudotime ordering of *Ccl3*^{wt} and *Ccl3*^{ko} ER-Hoxb8 neutrophils isolated from the blood and tumor-bearing lungs of mice with orthotopic KP1.9 tumors by flow cytometry, as $CD45.1^{+}CD11b^{+}Ly6G^{+}$ cells. n = 5-6 mice per group.

(b) KP1.9 tumor cells were injected i.v in mice for *in vivo* fate mapping experiments of *Ccl3*^{wt} and *Ccl3*^{ko} ER-Hoxb8 neutrophils. At day 18 post-injection, tumor burden of mice with *Ccl3*^{wt} or *Ccl3*^{ko} Hoxb8 neutrophils was measured by lung weight as a proxy. n = 11-12 mice per group. Unpaired t-test.

(c) Proportion and abundance of SiglecF^{hi} in *Ccl3*^{wt} or *Ccl3*^{ko} ER-Hoxb8 TANs *in vitro*, cultured for one day in TME-sup and measured by flow cytometry. Abundance refers to absolute cell counts. n = 3-6 replicates per group. Unpaired t-test.

(d) Schematic of *Ccl3*^{wt} or *Ccl3*^{ko} ER-Hoxb8 neutrophil progenitors cultured for three days in TME-sup or control differentiation media (left). Proportion of SiglecF^{hi} neutrophils was measured by flow cytometry at day 3 (middle). n = 3 replicates in control group and n = 8 replicates in TME-sup group. Relative expression of *Ccl3* in MACS-sorted Ly6G^{+} neutrophils at day 3 of culture was quantified by qPCR (right). n = 6 replicates per group from 2 independent sorts. Ordinary one-way ANOVA.

(e) Proportion of SiglecF^{hi} in *Ccl3*^{wt} or *Ccl3*^{ko} (*Ccl3* sg1-edited or *Ccl3* sg2-edited) ER-Hoxb8 TANs *in vitro*, cultured for three days in TME-sup and measured by flow cytometry at day 3. n = 4 replicates per group. Ordinary one-way ANOVA.

(f) Average expression of *Ccr1*/*CCR1, Ccr5*/*CCR5*, and *Ccr4/CCR4* in various immune cells of multiple mouse and human tumors (dataset shown in Figures 2-3 and Table S1-2).

(g) Proportion of CCR1^{+} and CCR5^{+} neutrophils *in vivo* in orthotopic KP1.9 lung tumors. n=5 mice per group.

(h) Proportion of CCR1^{+} (left) and SiglecF^{hi} (right) in *Ccr1*^{wt} or *Ccr1*^{ko} (*Ccr1* sg1-edited or *Ccr1* sg2-edited) ER-Hoxb8 TANs *in vitro*, cultured for three days in TME-sup and measured by flow cytometry at day 3. n = 4-7 replicates per group. Ordinary one-way ANOVA.

(i) Schematic of *Ccl3*^{wt} or *Ccl3*^{ko} ER-Hoxb8 neutrophil progenitors cultured for three days in TME-sup or control differentiation media (left). Relative expression of *Bcl2l1* in MACS-sorted Ly6G^{+} neutrophils at day 3 of culture was quantified by qPCR (right). n = 6-9 replicates per group from 2-3 independent sorts. Ordinary one-way ANOVA.

(j) Relative abundance of SiglecF^{hi} *Ccl3*^{wt} or SiglecF^{hi} *Ccl3*^{ko} ER-Hoxb8 TANs *in vitro*, cultured for three days in TME-sup with increasing concentrations (0 nM, 0.1 nM, 10 nM and 100 nM) of the H3-mimetic drugs A-1331852 or Navitoclax and measured by flow cytometry at day 3. Abundance refers to absolute cell counts relative to average *Ccl3*^{wt} counts with 0nM drug. n = 4 replicates per group. Ordinary one-way ANOVA.

Box and whiskers plot (min to max) are presented in (a), (j). Bar graphs in (b), (c), (g), (h) show mean ± SEM, and in (d), (e), (i) show median. **p<0.01, ***p<0.001, ****p<0.0001, ns, non significant.

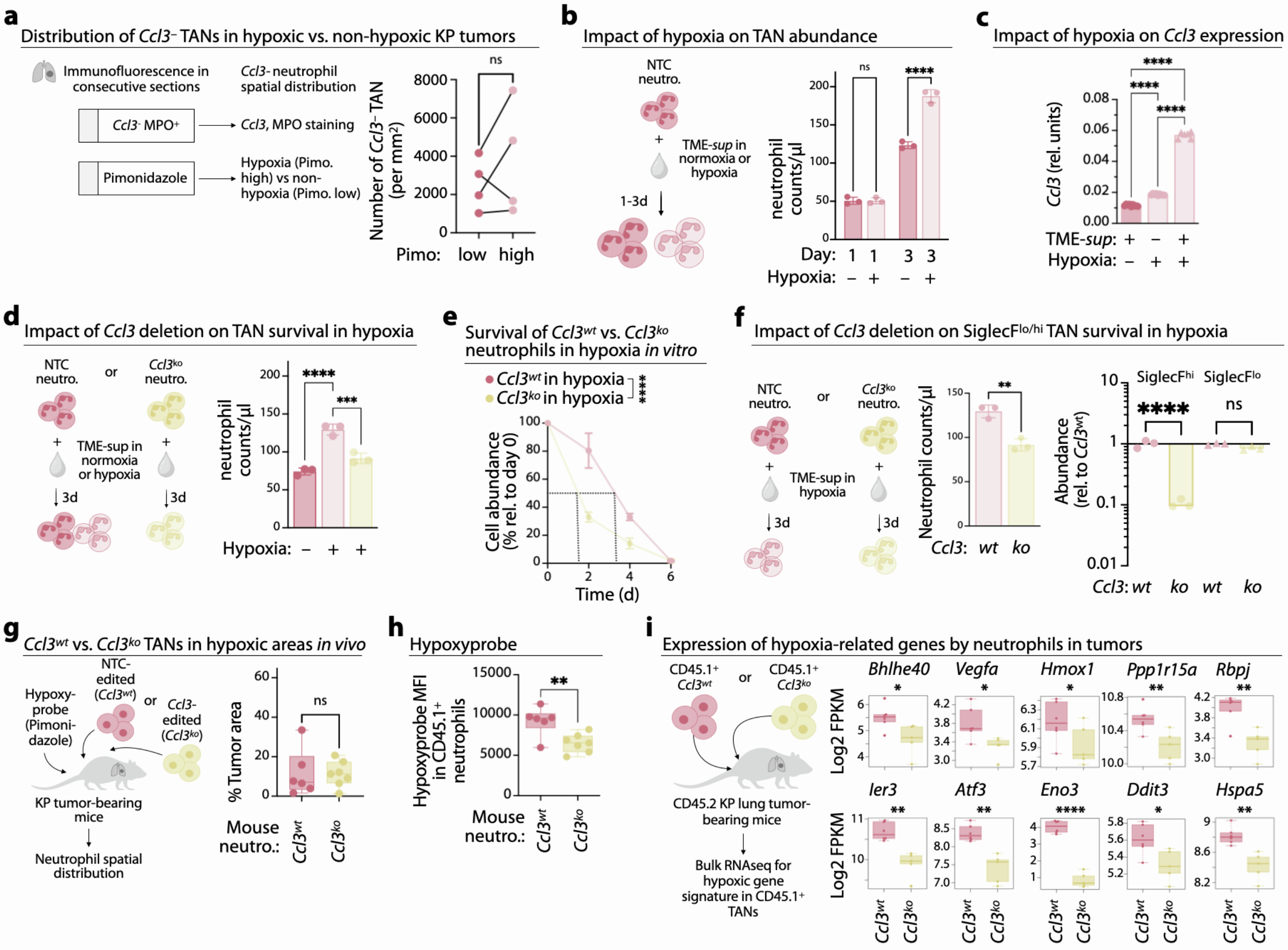

Figure S9: Hypoxic tumor niches promote the acquisition of the CCL3^{hi} neutrophil state, related to Figure 7.

(a) Schematic of experimental workflow. Due to limitations of the RNAscope workflow used to detect *Ccl3* transcripts, pimonidazole staining was performed on adjacent tissue sections (left). Absolute numbers of *Ccl3*$^-$ MPO$^+$ neutrophils per mm^2 in pimo^{lo} versus pimo^{hi} areas (right), as measured by immunofluorescence staining or RNAscope in consecutive sections. Pimonidazole was used as a probe for hypoxia (hypoxyprobe). n = 4 mice. Paired t-test.

(b) Schematic of ER-Hoxb8 neutrophil culture in TME-sup under either hypoxic or normoxic conditions (left). The abundance of ER-Hoxb8 TANs in these conditions was measured by flow cytometry at day 1 and 3 of culture (right). Abundance refers to absolute cell counts. n = 3 replicates per group; representative of two independent experiments. Two-way ANOVA.

(c) Relative expression of *Ccl3* in MACS-sorted Ly6G$^+$ ER-Hoxb8 neutrophils cultured for three days either in TME-sup or normal differentiation medium, and either under hypoxic or normoxic conditions. *Ccl3* expression was quantified by qPCR. n= 6 replicates per group from two independent sorts. Ordinary one-way ANOVA.

(d) Schematic of *Ccl3*^{wt} or *Ccl3*^{ko} ER-Hoxb8 neutrophil culture in TME-sup for three days under either hypoxic or normoxic conditions (left). The abundance of ER-Hoxb8 TANs in these conditions was measured by flow cytometry at day 3 (right). Abundance refers to absolute cell counts. n= 3 replicates per group. Ordinary one-way ANOVA.

(e) Fold change in *Ccl3*^{wt} or *Ccl3*^{ko} ER-Hoxb8 TAN abundance relative to day 0 after three-day culture in TME-sup under hypoxic conditions for TAN differentiation, followed by flow cytometric analysis at days 2, 4 and 6 to assess survival. Abundance refers to absolute cell counts. n = 3 replicates per group. Two-way ANOVA.

(f) Schematic of *Ccl3*^{wt} or *Ccl3*^{ko} ER-Hoxb8 TANs cultured *in vitro* for three days in TME-sup under hypoxic conditions and measured by flow cytometry at day 3 (left). Absolute TAN counts (middle) and relative abundance of SiglecF^{lo} and SiglecF^{hi} TANs (right), calculated as absolute cell counts relative to average *Ccl3*^{wt} counts, are shown. n = 3 replicates per group. Ordinary one-way ANOVA.

(g), (h) Schematic of spatial distribution of *Ccl3*^{wt} or *Ccl3*^{ko} ER-Hoxb8 TANs *in vivo* five days after their transfer (g-left). Tumor area within whole lung tissue, based on H&E staining, was quantified (g-right). Median Fluorescence Intensity (MFI) of the hypoxyprobe (pimonidazole) in *Ccl3*^{wt} or *Ccl3*^{ko} ER-Hoxb8 TANs *in vivo*, determined by immunofluorescence staining and quantified using Qupath software (h). n = 6-7 mice per group. Unpaired t-test.

(i) Bulk RNAseq analysis of *Ccl3*^{wt} and *Ccl3*^{ko} ER-Hoxb8 neutrophils isolated from KP1.9 lung tumors. *Ccl3*^{wt} or *Ccl3*^{ko} ER-Hoxb8 progenitors (CD45.1^{+} Cas9/GFP) were transferred i.v. in CD45.2^{+} Cas9/GFP recipients with orthotopic KP1.9 lung tumors and isolated by flow cytometry as CD45.1^{+} CD11b^{+} Ly6G^{+} cells. Log expression of hypoxia-related genes is shown. n = 5-6 mice per group. Wilcoxon test.

Box and whiskers plot (min to max) are presented in (g), (h), (i). Graphs in (b), (d), (e), (f-middle) show mean ± SD, and in (c), (f-right) show median. *p<0.05, **p<0.01, ***p<0.001, ****p<0.0001, ns, non significant.

Supplementary Tables and table titles

Table S1: scRNAseq datasets of human cancers, related to Figure 2.

Pan-cancer analysis (human)

Cancer type	Dataset	# patients
ESCC	Zhang et al.	58
HNSCC (1)	Bill et al.	52
HNSCC (2)	Kurten et al.	18
NSCLC (1)	Zilionis et al.	7
NSCLC (2)	Salcher et al.	17
PDAC	Wang et al.	5
		157

Table S2: scRNAseq datasets of murine tumor models, related to Figure 3.

Pan-cancer analysis (mouse)

Tumor site	Tumor model	Dataset	# mice
Glioma	IDH1	Alghamri et al.	2
Liver	*Nras*^{G12D}/*Pten*^{KO}	Ramirez et al.	1
Lung (1)	*Kras*^{LA1}	Prieto et al.	4
Lung (2)	KPT	Gonzalez et al.	4
Lung (3)	KP1.9	Zilionis et al.	2
Mammary (1)	MMTV-PyMT	Ramos et al.	2
Mammary (2)	4T1	Li et al.	13
Pancreas	KC, KPC	Caronni et al.	11
			39

Supplemental References

1. Zilionis, R., Engblom, C., Pfirschke, C., Savova, V., Zemmour, D., Saatcioglu, H.D., Krishnan, I., Maroni, G., Meyerovitz, C.V., Kerwin, C.M., et al. (2019). Single-Cell Transcriptomics of Human and Mouse Lung Cancers Reveals Conserved Myeloid Populations across Individuals and Species. Immunity *50*, 1317–1334.e10. https://doi.org/10.1016/j.immuni.2019.03.009.

2. Bill, R., Wirapati, P., Messemaker, M., Roh, W., Zitti, B., Duval, F., Kiss, M., Park, J.C., Saal, T.M., Hoelzl, J., et al. (2023). CXCL9:SPP1 macrophage polarity identifies a network of cellular programs that control human cancers. Science *381*, 515–524. https://doi.org/10.1126/science.ade2292.

3. Wang, L., Liu, Y., Dai, Y., Tang, X., Yin, T., Wang, C., Wang, T., Dong, L., Shi, M., Qin, J., et al. (2023). Single-cell RNA-seq analysis reveals BHLHE40-driven pro-tumour neutrophils with hyperactivated glycolysis in pancreatic tumour microenvironment. Gut *72*, 958–971. https://doi.org/10.1136/gutjnl-2021-326070.

4. Wu, Y., Ma, J., Yang, X., Nan, F., Zhang, T., Ji, S., Rao, D., Feng, H., Gao, K., Gu, X., et al. (2024). Neutrophil profiling illuminates anti-tumor antigen-presenting potency. Cell *187*, 1422–1439.e24. https://doi.org/10.1016/j.cell.2024.02.005.

5. Kürten, C.H.L., Kulkarni, A., Cillo, A.R., Santos, P.M., Roble, A.K., Onkar, S., Reeder, C., Lang, S., Chen, X., Duvvuri, U., et al. (2021). Investigating immune and non-immune cell interactions in head and neck tumors by single-cell RNA sequencing. Nat. Commun. *12*, 7338. https://doi.org/10.1038/s41467-021-27619-4.

6. Zhang, X., Peng, L., Luo, Y., Zhang, S., Pu, Y., Chen, Y., Guo, W., Yao, J., Shao, M., Fan, W., et al. (2021). Dissecting esophageal squamous-cell carcinoma ecosystem by single-cell transcriptomic analysis. Nat. Commun. *12*, 5291. https://doi.org/10.1038/s41467-021-25539-x.

7. Kim, N., Kim, H.K., Lee, K., Hong, Y., Cho, J.H., Choi, J.W., Lee, J.-I., Suh, Y.-L., Ku, B.M., Eum, H.H., et al. (2020). Single-cell RNA sequencing demonstrates the molecular and cellular reprogramming of metastatic lung adenocarcinoma. Nat. Commun. *11*, 2285.

8. Franken, A., Bila, M., Mechels, A., Kint, S., Van Dessel, J., Pomella, V., Vanuytven, S., Philips, G., Bricard, O., Xiong, J., et al. (2024). CD4+ T cell activation distinguishes response to anti-PD-L1+anti-CTLA4 therapy from anti-PD-L1 monotherapy. Immunity *57*, 541–558.e7.

9. Kang, M., Gulati, G.S., Brown, E.L., Qi, Z., Avagyan, S., Armenteros, J.J.A., Gleyzer, R., Zhang, W., Steen, C.B., D'Silva, J.P., et al. (2025). Improved reconstruction of single-cell developmental potential with CytoTRACE 2. Nat. Methods *22*, 2258–2263.

10. Ng, M.S.F., Kwok, I., Tan, L., Shi, C., Cerezo-Wallis, D., Tan, Y., Leong, K., Calvo, G.F., Yang, K., Zhang, Y., et al. (2024). Deterministic reprogramming of neutrophils within tumors. Science *383*, eadf6493. https://doi.org/10.1126/science.adf6493.

11. Gungabeesoon, J., Gort-Freitas, N.A., Kiss, M., Bolli, E., Messemaker, M., Siwicki, M., Hicham, M., Bill, R., Koch, P., Cianciaruso, C., et al. (2023). A neutrophil response linked to tumor control in immunotherapy. Cell *186*, 1448–1464.e20. https://doi.org/10.1016/j.cell.2023.02.032.
